# Foreign Direct Investments, Renewable Electricity Output, and Ecological Footprints: Do Financial Globalization Facilitate Renewable Energy Transition and Environmental Welfare in Bangladesh?

**DOI:** 10.1007/s10690-021-09335-7

**Published:** 2021-04-26

**Authors:** Muntasir Murshed, Mohamed Elheddad, Rizwan Ahmed, Mohga Bassim, Ei Thuzar Than

**Affiliations:** 1 School of Business and Economics, North South University, Dhaka, Bangladesh; 2 Department of Management, Huddersfield Business School, University of Huddersfield, Queensgate, Huddersfield, HD13DH UK; 3 Birmingham Business School, University of Birmingham, Birmingham, UK; 4 School of Humanities, The University of Buckingham, Buckingham, UK; 5 Cardiff Business School, University of Cardiff, Cardiff, UK

**Keywords:** Renewable energy, Renewable electricity, FDI, Ecological footprints, Pollution haven hypothesis, EKC hypothesis, Structural breaks, Q4, Q42, Q43, F3

## Abstract

Phasing out fossil fuel dependency to adopt renewable energy technologies is pertinent for both ensuring energy security and for safeguarding the well-being of the environment. However, financial constraints often restrict the developing countries, in particular, from undergoing the renewable energy transition that is necessary for easing the environmental hardships. Against this background, this study makes a novel attempt to evaluate the impacts of FDI inflows on enhancing renewable energy use and attaining environmental sustainability in Bangladesh between 1972 and 2015. Using the autoregressive distributed lags with structural break approach to estimate the short- and long-run elasticities, it is found that FDI inflows enhance the share of renewable electricity output in the total electricity output levels of the country. Besides, FDI inflows are also evidenced to directly hamper environmental quality by boosting the ecological footprints figures of Bangladesh. Hence, it can be said that FDI promotes renewable electricity generation in Bangladesh but transforms the nation into a pollution haven. However, although FDI inflows cannot directly reduce the ecological footprints, a joint ecological footprint mitigation impact of FDI inflows and renewable electricity generation is evidenced. Besides, the findings also verify the authenticity of the Environmental Kuznets Curve hypothesis in Bangladesh’s context. Therefore, economic growth can be referred to as being both the cause and the panacea to the environmental problems faced by Bangladesh. These results, in a nutshell, calls for effective measures to be undertaken for attracting the relatively cleaner FDI in Bangladesh whereby the objectives of renewable energy transition and environmental sustainability can be achieved in tandem. In line with these findings, several appropriate financial globalization policies are recommended.

## Introduction

As an economy grows, it is expected that its overall energy demand would increase in tandem (Bashir et al., 2020; Talbi et al., 2020). Accordingly, the paramount importance of ensuring energy security for sustaining economic welfare across both the developing and the developed countries has been broadly highlighted within the development economics narrative (Tang et al., 2016). Although a reliable supply of energy is often asserted to be a pre-requisite to fostering the overall development processes, often a trade-off between higher economic prosperity and lower environmental well-being can be witnessed (Murshed et al., 2021a; Jamel & Derbali, 2016). This is because, predominantly, fossil fuels are utilized to meet the global energy demand whereby combustion of these energy resources exerts adverse evironmental issues. For instance, fossil fuel consumption decisions trigger the emissions of pollutants into the atmosphere; thus, causing the environmental quality to deteriorate (Hanif et al., 2019). Conversely, utilizing renewable energy resources as an alternative has been hypothesized to improve the state of the environmental attributes (Murshed, 2021a). Hence, it is pertinent to unearth the possible means through which fossil fuel dependency across the globe can be mitigated to ensure complementarity between economic and environmental development.

Electricity is the most prominently utilized source of energy employed to produce the national outputs. However, countries, and in particular the developing ones, have traditionally relied on the indigenous primary fossil fuel supplies for electricity-generation purposes. Besides, these nations have also been overwhelmingly dependent on imported fossil fuels for generating electricity (Murshed et al., 2020a; Murshed & Tanha, 2020). As a result, such fossil fuel dependency has led to the surge in global gas emissions which, in turn, has gone on to aggravate the global environmental quality as well (Covert et al., 2016). Thus, replacing the traditional fossil fuels with relatively modern and cleaner alternatives is believed to be an effective means of restoring environmental harmony, worldwide (Nathaniel et al., 2021a; Murshed, 2021b). In this regard, it is often hypothesized that the augmentation of renewable energy into the global energy mix can facilitate economic growth without marginalizing the environmental quality worldwide (IRENA, 2019). Likewise, renewable energy adoption has also been linked with climate change mitigation since combustion of these resources do not emit greenhouse gases into the atmosphere (IRENA, 2018).

Apart from environmental betterment, the overall benefits of enhancing the global Renewable Electricity Output (REO) levels can be embodied in multidimensional forms. For instance, the employment of renewable electricity within the manufacturing industries is anticipated to bridge the acute power shortages that are responsible for below-par levels of industrial outputs (Beier et al., 2019). On the other hand, large-scale renewable electricity supply can also be conducive to amplify the access to off-grid electricity for agricultural cultivation (Guerin, 2019). Besides, higher REO levels can be efficient in mitigating energy poverty by resolving the low electrification rates woes of the developing nations, in particular (Wang et al., 2019). Moreover, the development of the renewable electricity sector could also reap dividends in the context of illuminating the rural communities; thus, improving the rural electrification rates to a large extent as well (Melkior et al., 2018). Additionally, hybrid off-grid renewable electric systems can complement the conventional in-grid electricity supplies to boost the overall reliability of the power supply (Odou et al., 2020). In this regard, decentralization of the traditional centralized power supply infrastructure particularly through the incorporation of renewable resources for off-grid electrification is hypothesized to be a futuristic solution to the energy crises faced by the underdeveloped global economies in particular (Moner-Girona et al., 2019). Furthermore, transitioning from non-renewable to renewable electricity generation can create employment opportunities to facilitate social development as well (Ram et al., 2020). Hence, keeping into consideration these environmental and non-environmental benefits, it is pertinent to identify the macroeconomic factors which can boost renewable electricity generation worldwide.

However, integrating renewable electricity into the national electricity-mix can be a cumbersome task, especially for the developing economies which are unable to rapidly undergo the Renewable Energy Transition (RET) phenomenon due to several limitations (Xue et al., 2021; Nathaniel et al., 2021b). For instance, finite and unreliable primary renewable energy supplies often inhibit the adoption of renewable electricity generation technologies within these economies. However, and more importantly, technological underdevelopment and poor energy infrastructures are considered as the central factors withstanding mass-scale generation of REO (Hille et al., 2020; Ogbonnaya et al., 2019). At the same time, since technological advancement and energy infrastructural development require hefty amounts of investment, both from the public and private sectors (Rafique et al., 2019), it is often not possible for the underdeveloped nations to finance such investments. Thus, financial constraint is an additional constraint faced by the developing nations which oblige them to be fossil fuel-dependent to a large extent. Besides, the significantly high start-up cost associated with the setup of renewable electricity generation plants has also played a role in sustaining the fossil fuel-dependency among developing countries (Abe et al., 2016; Kabel & Bassim, 2020). Therefore, reducing the financial constraints to overcome the technological and infrastructural barriers to undergoing RET is critically important for these nations.

In this regard, Foreign Direct Investment (FDI) inflows into the energy sectors of the underdeveloped nations can depict noteworthy implications for facilitating the RET phenomenon (Rahman, 2015; Hübler and Keller, 2010; Er et al., 2018). Linking FDI inflows to the integration of renewable electricity into the grid, and also to environmental betterment, the United Nations’ 2030 agenda of Sustainable Development Goals (SDG) calls for substantially elevating the REO share in the global electricity-mix, particularly through the mobilization of external financial resources such as FDI. Attracting FDI into the relatively poor economies, apart from attaining socioeconomic growth overall, is anticipated to promote technological and knowledge spillovers that can effectively bridge the technological gap inhibiting RET (Liu et al., 2016).

Against this milieu, this study aims to evaluate the impacts of FDI inflow on Bangladesh’s REO share in the aggregate electricity production figures and environmental quality between 1972 and 2015. Bangladesh is a fast-emerging South Asian economy that has registered tremendous growth achievements over the years. The nation has recently eclipsed the criteria of twin graduation to be classified as a lower-middle-income and developing country. Bangladesh is also a member of the Next Eleven countries whereby the nation is expected to experience robust degrees of economic growth in the years to come. As a result, meeting the future energy demand is a major challenge for the government. At the same time, the nation’s traditional reliance on fossil fuels-based electricity generation strategies to meet its energy demand could also go on to jeopardize environmental sustainability in Bangladesh. Thus, keeping Bangladesh’s energy security and environmental sustainability into cognizance, evaluating the macroeconomic factors which can facilitate RET in Bangladesh is extremely important. Besides, the outcomes from this study can be expected to assist in efficient policymaking concerning the nation’s achievement of the SDG by 2030. Furthermore, this study is also relevant from the perspective that Bangladesh has not succeeded in achieving its national target of generating at least 10% of the total electricity output from renewable sources by 2020. On the other hand, Bangladesh is also one of the countries under China’s Belt and Road Initiative (BRI). As a result, the nation can expect to receive hefty amounts of Chinese outward-FDI which, in turn, can be anticipated to have a sizeable impact on the prospects of enhancing renewable power generation figures of Bangladesh. Most importantly, this study can be a standpoint for Bangladesh to phase out its traditional fossil fuel-dependency issues to develop the renewable energy sector. Moreover, this current study is also timely from the perspective that the Asian Development Bank has forecasted Bangladesh to outperform the other South Asian economies in respect of economic recovery following the Covid-19 pandemic (ADB, 2020). Hence, it can be expected that the economic activities in Bangladesh are likely to surge from 2021 onwards which would also boost the domestic energy demand. Hence, enhancing REO can help facilitate Bangladesh's post-Covid-19 economic recovery.

This study contributes to the literature in four-fold. Firstly, to the best of the authors’ knowledge, this is the seminal study that links FDI inflows to changes in the REO shares in the context of Bangladesh. Several preceding studies have assessed the impacts of such foreign financial inflows on renewable energy consumption, mostly using panel data sets including data of multiple countries including Bangladesh. However, the country-specific analysis in the case of Bangladesh is yet to be extensively documented in the literature. This current study aims to bridge this gap in the literature. Secondly, this study also emphasizes the joint impacts of FDI inflows and REO on the environmental quality in Bangladesh. The preceding studies have primarily explored the individual effects and have overlooked the joint impacts. However, for efficient policymaking purposes, unearthing the possible joint impacts is important. Thirdly, as opposed to the conventional approach of using Carbon dioxide (CO_2_) emissions to proxy for environmental quality in Bangladesh, this study considers the ecological footprints to measure the changes in the quality of the environment in response to incoming FDI. Compared to CO_2_ emissions, the ecological footprint is a more comprehensive measure of environmental quality (Nathaniel et al., 2019). Very few country-specific studies on Bangladesh have assessed the environmental impacts using the ecological footprint figures. Lastly, the empirical analysis is carried out using recently developed econometric tools that are robust to accounting for the structural break issues in the data. Not many existing studies have controlled for the structural break issues to model renewable electricity production and environmental quality in Bangladesh’s context.

The remainder of the study is structured as follows. Section 2 provides a brief overview of the electricity sector in Bangladesh. A study of the relevant theoretical and empirical literature is presented in Sect. 3. Section 4 explains the empirical model and describes the data set used in conducting the econometric analyses while Sect. 5 notes down the methodology tapped to answer the research questions. Section 6 reports and discusses the results found from the statistical tests. Finally, Sect. 7 provides the concluding remarks.

## An Overview of Bangladesh’s Electricity Sector

**Fig. 1:**
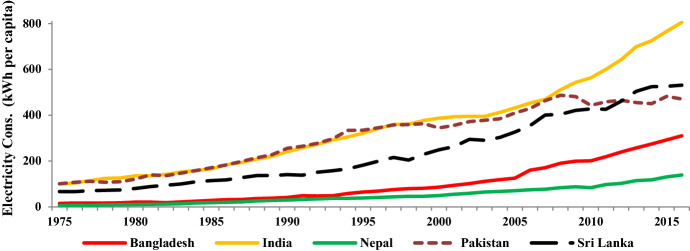
Trends in electricity consumption per capita across South Asia. *Source:* Author's compilation from the World Development Indicators database (World Bank, 2,020)

Ever since its independence in 1971, the economy of Bangladesh has undergone numerous waves of structural transformations (Murshed, 2021c). Consequently, the nation has gradually transformed its traditional agrarian economy into a modern industrialized one. At the same time, the total electricity demand of the nation has persistently surged over time. Although the local electricity demand could not be matched with the indigenous electricity outputs, the absolute volumes of per capita electricity consumption have gone up from being around 15 kilowatt-hours in the early 1970s to more than 310 kilowatt-hours by the end of 2016; thus, indicating a 21-fold rise in the per capita electricity consumption figures (World Bank, 2020). Figure 1 compares Bangladesh’s per capita electricity consumption figures with several of its South Asian neighbors. It is evident from the graphical projections that the per capita electricity consumption figures of Bangladesh, although hovering above those of Nepal, have always lagged behind the corresponding figures of India, Pakistan, and Sri Lanka. Hence, this clearly certifies Bangladesh’s acute electricity supply concerns which, to an extent, have pinned down the nation’s true growth potentials.

**Fig. 2:**
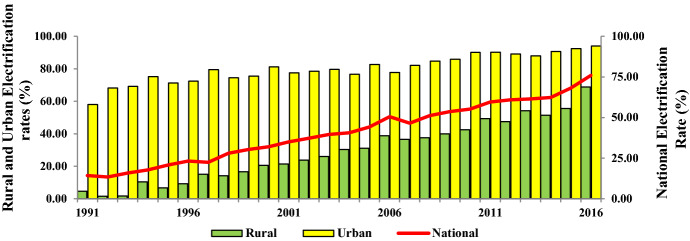
Trends in access to electricity in Bangladesh. *Note* National, Rural, and Urban electrification rates are expressed as percentages of national, rural, and urban populations, respectively. *Source*: Author's compilation from the World Development Indicators database (World Bank, 2,020)

Figure 2 provides a graphical illustration of the national, rural, and urban electrification rates in Bangladesh. It can be seen that Bangladesh has managed to substantially improve its national electrification rates from 8.5% in the early 1990s to almost 76% by the end of 2016. However, a clear rural–urban divide can be perceived from the corresponding rural and urban electrification rates. Between 1990 and 2016, the urban electrification rates have gone up by 15-fold while the rural electrification rates registered a mere 1.5-fold increment. A particular reason behind these contrasting phenomena can be credited to the relatively lower access to in-grid connectivity across rural regions of Bangladesh. Nonetheless, a gradual convergence between the rural and urban electrification rates is evident; though the gap is still a significant one. Under such circumstances, decentralized off-grid electrification, especially across rural neighborhoods, can be a credible means of mitigating this disparity. Thus, RET in Bangladesh is critically important in this regard.

**Fig. 3:**
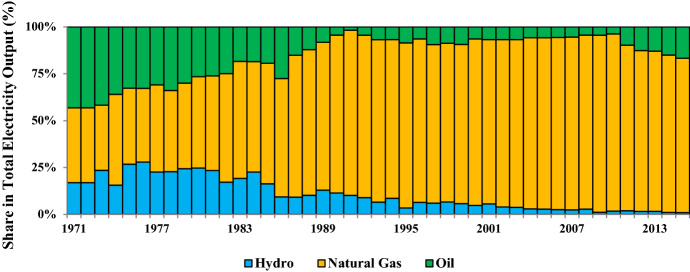
Major sources of electricity outputs in Bangladesh. *Source*: Author's compilation from the World Development Indicators database (World Bank, 2,020)

**Fig. 4:**
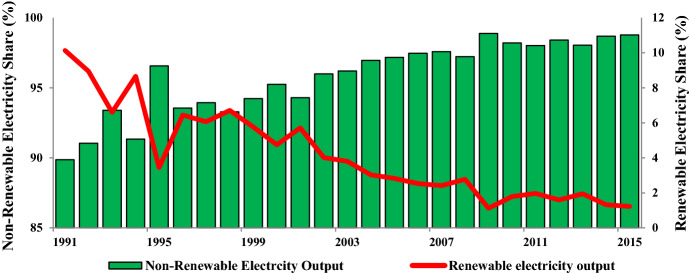
Renewable and non-renewable electricity output shares. *Source*: Author's compilation from the World Development Indicators database (World Bank, 2,020)

The aggregate electricity output of Bangladesh has mostly been generated from fossil fuels, domestic natural gas, and imported crude oil, in particular. Besides, a nominal amount of electricity is also generated from coal. Figure 3 depicts the shares of key primary energy inputs in the aggregate electricity output of Bangladesh. It can be seen that in the early 1970s, the share of imported oil outweighed the shares of natural gas and hydropower. However, following the exogenous shocks to world oil prices, the government decided to supply the indigenous natural gas at outrageously subsidized prices to lessen the nation’s predominant imported-oil dependency for power generation purposes. As a consequence, both the shares of imported oil and hydropower have substantially reduced over the years. Between 1971 and 2015, the shares of these energy resources respectively fell from 43.11% and 16.95% to 16.38% and 0.96%. On the other hand, the share of natural gas surged from 39% to more than 80%. The significant decline in the hydropower shares reveals Bangladesh’s failure to increase the REO, particularly due to technological and infrastructural constraints. The declining trends in the REO shares, between 1990 and 2015, can be understood from Fig. 4. However, the acute natural gas supply crunch at present imposes ominous threats for the nation’s energy security in the future. Although the nation aims to complement the natural gas-fired electricity outputs by enhancing the share of coal-fired electricity in the aggregate electricity output figures (The Daily Star 2019, November 07), such energy sector reforms are not conducive to simultaneously ensuring economic growth and environmental sustainability. Under such circumstances, enhancing the REO shares should be a priority for the government.

Therefore, it is evident from these graphical illustrations that Bangladesh has tradionally been reliant on both domestic and imported fossil fuels for electricity generation purposes. However, the nation has not managed to reduce this monotonic fuel dependency which, in turn, has resulted in adverse environmental consequences. Besides, the graphs also portrayed a picture of the rural–urban gap in energy poverty which can be curbed through off-grid renewable electricifation across the rural regions of Bangladesh. Hence, keeping in to consideration Bangladesh’s energy security and environmental sustainability, it is necessary to examine the channels through which RET in Bangladesh can be expedited heading towards the deadline of the SDG agenda of the United Nations.

## Literature Review

This section is divided into two sub-sections. Firstly, the theoretical framework in the context of the effects of FDI inflows on REO and environmental sustainability is put forward. Secondly, the corresponding empirical evidence documented in the literature is summarized.

### Theoretical Framework

In the context of developing nations, financial constraint is a major growth-inhibiting concern. At the same time, technological limitations and infrastructural backwardness limit the prospects of undergoing RET and safeguarding environmental sustainability in tandem. Under such circumstances, attracting FDI can be expected to lessen the financial constraints and simultaneously overcome the other barriers inhibiting RET and environmental development (Murshed et al., 2021b; Liu et al., 2016). Besides, mitigating CO_2_ and other harmful emissions has become a global agenda following the ratification of the Paris Agreement (Chaudhry et al., 2020) by several of the world economies including Bangladesh. Thus, in this regard, is it important to reduce the use of fossil fuels and significantly enhance renewable energy use.

Renewable electricity generation is a relatively technology-intensive process. Thus, technological innovation is a pre-requisite to initiate the RET phenomenon (Ben Jebli & Kahia, 2020; Dagoumas & Koltsaklis, 2019; Eleftheriadis & Anagnostopoulou, 2015). However, the poor state of technology in the majority of the developing nations, including Bangladesh, often bottlenecks the prospects of phasing out the dependency on fossil fuel consumption to utilize renewable energy alternatives (Murshed et al., 2020b; Murshed, 2020a). Besides, the generation, transmission, and distribution of electricity generated from renewable resources require the energy infrastructure to be advanced which, once again, is a RET-inhibiting constraint faced by the developing nations. These limitations can be expected to be overcomed through a knowledge spillover impact associated with globalization, especially in the form of FDI inflows (Padhan et al., 2020; Hoang et al., 2021). In reference to the absorptive capacity hypothesis (Navas, 2019), FDI inflows can exert a positive spillover impact to promote renewable energy adoption within the host economies. Consequently, FDI inflows can induce renewable energy augmenting effect by discouraging the use of non-renewable fossil fuels in the host economies (Narayan and Doytch, 2016). At the same time, incoming FDI can also be expected to foster the development of the host economy’s renewable energy sector growth by upgrading the poor energy infrastructure (Ji & Zhang, 2019). Conversely, FDI inflows, especially the dirty FDI, can also exert dampening impacts on REO. This is because the relatively unclean FDI is often targeted at economies that are predominantly fossil fuel-intensive. Under such circumstances, such FDI inflows can boost non-renewable energy use to hinder the RET phenomenon across the host nations (Cole et al., 2006; Shahbaz et al., 2019). Hence, it is ideal for the developing nations to attract clean FDI, especially for the development of their respective renewable energy sector.

On the other hand, it is often recommended to pursue green economic and financial policies to ensure environmental welfare (Karyawati et al., 2020; Kovilage, 2020). In line with this notion, FDI inflows have been acknowledged to exert both positive and negative environmental impacts on the host nations. The adverse environmental effects associated with FDI inflows is explained using the theoretical underpinnings of the ‘*pollution haven hypothesis*’ (Nasil et al., 2019; Doytch, 2020). This hypothesis asserts that inflows of dirty FDI tend to boost the use of unclean energy resources which, in turn, stimulate the emission of greenhouse gases to degrade the overall quality of the environment in the host economies (Solarin et al., 2017). In contrast, the ‘*pollution halo hypothesis*’ postulates in favor of FDI inflows exerting positive environmental impacts within the host economies (Liu & Xu, 2021). In this regard, clean FDI is hypothesized to green the production and consumption processes within the host nations to improve the quality of the environment. Furthermore, since renewable energy use is believed to be inextricably linked to environmental development (Dogan et al., 2020; Nathaniel et al., 2020; Adedoyin et al., 2020; Sharma et al., 2021), a joint environmental impact of FDI inflows and renewable energy consumption can also be expected (Zhang & Zhou, 2016).

### Empirical Evidence

#### The Literature on FDI Inflows and Renewable Energy Use

The impacts of FDI inflows on renewable energy use have received a significant amount of mention in the recent literature. In a study on 21 African economies, FDI inflows were found to be associated with higher shares of renewable energy in the aggregate energy consumption figures. Based on these findings, the author suggested that FDI flowing into the African economies is directed at industries that are relatively intensive in the use of renewable energy resources. Thus, a technological spillover impact of FDI can be hypothesized. In contrast, Murshed (2020a) found no conclusive evidence of FDI inflows influencing the renewable energy consumption shares in the context of 71 low, middle and high-income countries. However, the author opined in favor of FDI elevating the level of renewable energy use only in the low-income countries. Similar results in the context of 4 Southeast Asian economies were put forward by Kumaran et al. (2020). In another recent study by Murshed (2020b), FDI inflows were referred to reduce renewable energy use in Bangladesh and five other South Asian economies. Moreover, the shares of renewables in the total final primary energy consumption figures were also found to be negatively impacted by higher inflows of FDI. As opposed to these findings, Lee (2013) found no statistical evidence of FDI inflows affecting renewable energy consumption across the Group of 20 (G20) economies. These different results in the abovementioned studies are expected considering the heterogeneity in respect of the income groups these countries belong to and also in respect of the disparities in the stringency of their environmental laws.

Now, shifting the focus from renewable primary energy resources to renewable electricity generation, Ankrah and Lin (2020) found statistical evidence of positive long-run associations between FDI inflows and the volume of renewable electricity produced in Ghana. On the other hand, Kilicarslan (2019) found incoming FDI to dampen renewable electricity outputs in a panel data analysis concerning Brazil, Russia, India, China, South Africa, and Turkey. Bento Cerdeira (2012) probed into the FDI-REO nexus in the context of Portugal and found evidence of unidirectional short-run causality running from REO to FDI. However, in the long-run, bidirectional causation between these variables was ascertained. In contrast, Mac Domhnaill and Ryan (2020) examined the effects of FDI inflows on the renewable electricity outputs in selected European economies and concluded that there is no significant effect. Based on the findings, it was asserted that FDI cannot facilitate a knowledge spillover effect on the European nations. Ahmad et al. (2019) found unidirectional short and long-run causalities to run from FDI inflows to REO in China. Based on the findings from most of these studies, it can be claimed that attracting FDI can be a solution to the low REO shares in developing countries. Likewise, Majid (2020) recommended the attraction of FDI into the Indian economy to enhance the nation's renewable electricity generation capacities by 2040. The authors also claimed that FDI inflows can help the Indian economy to get over its predominant reliance on coal for electricity generation purposes. Besides, Kutan et al. (2018) showed how FDI can finance renewable energy projects in major emerging market economies that have traditionally faced financial constraints that impeded the development of the renewable energy sector in the respective economies. Similarly, Golusin et al. (2010) opined that channeling FDI to the electricity sector of Serbia can be effective in boosting the nation’s REO shares in the total electricity outputs.

#### The Literature on FDI Inflows and Environmental Quality

Although inflow of FDI is assumed to synthesize economic growth in the host nation (Nguyen et al., 2020; Su et al., 2019), it can also exert environmental effects. Hence, several preceding studies have probed into the FDI inflow-environmental quality nexus. However, the impacts of FDI inflows on the quality of the environment have received equivocal mentions in the literature. While a plethora of the existing studies claimed greater financial globalization in the form of higher FDI inflows to be a pre-requisite to ensuring technological spillover-induced environmental welfare within the host nations, several studies have also advocated against this statement. Among the studies that have opined in favor of FDI inflows generating favorable environmental outcomes, Saud et al. (2019) found that FDI flowing into 59 countries under China’s Belt and Road Initiative (BRI) reduces the per capita CO_2_ emission figures. In another recent study concerning 90 BRI economies, Ahmad et al. (2020) also found similar environmental impacts associated with FDI inflows. Similarly, Rafindadi et al. (2018) inferred that FDI flowing in the member states under Gulf Cooperation Council tends to curb their CO_2_ emission levels. Besides, the author also found statistical evidence of joint positive impacts of FDI inflows and international trade on environmental quality; hence, these results also implied an indirect impact of FDI on the environment. In another study on a panel of 188 global economies, Shao (2018) remarked that FDI inflows improve environmental quality by reducing the carbon intensities. The results were homogeneous for the full sample as well as for the low-, middle- and high-income groups. Similar results were reported in the context of selected Southeast Asian economies by Ansari et al. (2019).

In a country-specific analysis, Abbasi and Riaz (2016) showed that financial development and FDI inflows boost CO_2_ emissions in Pakistan. The authors added in their conclusions that attracting FDI, although beneficial for catalyzing the growth of the Pakistan economy, does not safeguard the nation’s environmental welfare. On the other hand, exploring the possible non-linear FDI-CO_2_ emission nexus for Brazil, Russia, India, China, South Africa, and Turkey, Abdouli et al. (2018) showed that FDI inflows beyond a threshold level reduce their CO_2_ emission levels. On the other hand, using data on 150 Chinese provinces, Jiang et al. (2018) also found that FDI inflows impose positive impacts on the environmental quality. The authors particularly claimed that FDI inflows exert technological spillover effects across the Chinese provinces which have enabled them to reduce the aggregate CO_2_ emissions in China. On the other hand, among the few studies that used ecological footprints to quantify environmental quality in this regard, Zafar et al. (2019) and Khan et al. (2020) found FDI inflows to be useful in reducing the ecological footprints to restore environmental harmony in the United States and India, respectively. Can et al. (2020) also assessed the impacts of FDI on ecological footprints in the context of 84 developing countries. However, the corresponding elasticity estimates, although depicting the desired negative sign, were found to statistically insignificant. Thus, the variations in the ecological footprint levels of these countries could not be explained by their respective FDI levels. Therefore, it is evident from all these aforementioned studies, except the one by Can et al. (2020), that FDI inflows improve environmental quality. As a result, these studies have certified the authenticity of the ‘*pollution halo hypothesis*.’

In contrast, a large number of studies have authenticated the ‘*pollution haven hypothesis*’ by highlighting the adverse environmental impacts associated with FDI inflows. In a study on 11 low-income, 53 middle-income, and 35 high-income countries, Shahbaz et al. (2015) found statistical evidence of FDI inflows degrading environmental quality by boosting the CO_2_ emissions. The findings were held for both the combined panel of 99 economies, as well as for the three sub-panels classified in terms of their respective income groups. Similarly, in a recent study, Abdo et al. (2020) found that FDI flowing into selected Arab nations resulted in higher CO_2_ emissions. Similar findings were reported by Shahbaz et al. (2018) for France. The authors asserted that although financial development and energy innovation lower CO_2_ emissions in France, incoming FDI inflows tend to boost the CO_2_ emission levels. In a similar study on 17 Middle Eastern and North African (MENA) countries, Abdouli and Hammami (2018) found contrasting impacts of FDI on CO_2_ emissions. The authors found that FDI inflows degrade environmental quality only in the context of the 12 Middle Eastern economies but in the case of the North African nations. Besides, Abdouli and Hammami (2017) concluded in favor of a positive correlation between FDI inflows and CO_2_ emissions for Kuwait, Marico, and Qatar. Recently, Murshed (2021d) concluded that FDI inflows deteriorate environmental quality within six South Asian economies including Bangladesh. The results showed that higher volumes of FDI enhanced the volumes of CO_2_, methane, nitrous oxide, and aggregate greenhouse gas emissions. Also, Nguyen et al. (2018) and (2019) found FDI inflows to trigger the CO_2_ emissions in the case of selected emerging economies.

Among the few studies using ecological footprints to proxy for environmental quality, Khan et al. (2019) found that the FDI flowing into Bangladesh and 53 other BRI economies contributed to higher ecological footprints. Similar results in the context of 47 Organization for Islamic Cooperation countries were reported by Ali et al. (2020). Similarly, in a study of the environmental issues in 44 BRI countries, Liu and Kim (2018) opined that FDI inflows are detrimental to the quality of the environment. Likewise, Chowdhury et al*.* (2020) examined the impacts of FDI inflows on the ecological footprint figures of 92 global economies. The study revealed that FDI causes environmental degradation by boosting ecological footprints. In another relevant study, Balsalobre-Lorente et al. (2019) found an inverted-U-shaped association between FDI inflows and ecological footprints, for Mexico, Indonesia, Nigeria, and Turkey. In line with the findings, the authors claimed that although FDI inflows initially degrade environmental quality, the effects get reversed beyond a certain level of FDI inflows. Conversely, Destek and Okumus (2019) asserted that at lower levels of FDI inflows the ecological footprints in 10 newly industrialized economies tend to decline; but the impact does not sustain at higher degrees of FDI inflows. Moreover, using CO_2_ emissions, carbon footprints, and ecological footprints as indicators of environmental pollution, Solarin and Al-Mulali (2018) found evidence of higher FDI inflows contributing to greater environmental pollution in developed economies, but not in the context of developing economies.

Based on the abovementioned theoretical frameworks and the corresponding empirical evidence, several hypotheses in the context of Bangladesh can be put forward:

##### Hypothesis 1

FDI inflows mitigate the constraints which inhibit the RET phenomenon.

##### Hypothesis 2

FDI inflows environmental impacts on the host economy.

##### Hypothesis 3

A joint environmental impact of FDI inflows and renewable energy use exists.

## Empirical Models and Data

In order to evaluate the impacts of FDIs on the REO responses of Bangladesh, this study follows Murshed (2020a) and considers a linear econometric model in which the REO share in Bangladesh’s total electricity output is expressed as a function of FDI inflows and other key control variables that have been theorized to affect the FDI inflow-REO nexus:

1 $${\varvec{lnREO}}_{{\varvec{t}}} = \varvec{ \delta }_{0} + \varvec{ \delta }_{1} {\varvec{lnFDI}}_{{\varvec{t}}} + \varvec{ \delta }_{2} {\varvec{lnEI}}_{{\varvec{t}}} + {\varvec{\delta}}_{3} {\varvec{lnGDPPC}}_{{\varvec{t}}} + \varvec{ \delta }_{4} {\varvec{lnGCF}}_{{\varvec{t}}} + \varvec{ \delta }_{5} {\varvec{lnOPEN}}_{{\varvec{t}}} + {\varvec{\delta}}_{6} {\varvec{lnOIL}}_{{\varvec{t}}} + {\varvec{\delta}}_{7} {\varvec{lnCO}}2_{{\varvec{t}}} + {\varvec{\varepsilon}}_{{\varvec{t}}} \user2{ }$$

where the subscript t denotes the time period, δ_i_ (i = 1, …, 7) represent the elasticity parameters to be estimated and ε is the random error term. All the variables are transformed into their natural logarithms for the ease of estimating the conditional bivariate elasticities. The dependent variable REO refers to the percentage shares of electricity generated from renewable resources in the aggregate electricity outputs of Bangladesh. FDI is the principal regressor of interest which denotes the total volume of FDI flowing into Bangladesh, measured in terms of constant 2010 US dollars. Besides, the econometric model is also controlled for the technological advancement within the Bangladesh economy. In this regard, a change in the level of technological advancement is proxied by the energy intensity level (denoted by lnEI) which is measured in terms of energy use (kilograms of oil equivalent) per unit of real GDP. A decline in the energy intensity level can be interpreted as an improvement in energy efficiency which, in turn, can be credited to advancement in technology and vice-versa. Technological innovation is said to enhance the stock of technology within the economy which can be expected to facilitate the integration of renewable energy in the national energy-mix of Bangladesh (Kumar & Agarwala, 2016). Also, the model is controlled for the growth of the Bangladesh economy. Economic growth is proxied by the real value of the per capita Gross Domestic Product (GDPPC), measured in constant 2010 US dollars. Economic growth plays a key role in the renewable energy transition process, whereby a rise in the level of economic growth can be anticipated to enhance the volume of REO (Tahvonen & Salo, 2001). The econometric model also controls for the level of capital investment in Bangladesh, measured using the level of Gross Capital Formation (GCF), measured in terms of constant 2010 US dollars. Since capital investments are more likely to happen in the industry sector, it is expected to increase the electricity demand in Bangladesh (Abokyi et al., 2018). Since Bangladesh predominantly generates electricity from fossil fuels, a rise in the value of the GCF is likely to reduce the share of REO.

OPEN denotes the trade openness index which expresses the total volume of Bangladesh’s exports and imports as a percentage of its GDP. It is hypothesized that greater openness to trade can cater to the inflow of relevant technical expertise and other inputs necessary for generating electricity from renewable energy resources (Omri & Nguyen, 2014). The inclusion of openness to trade data into the model is justified in the sense that Bangladesh imports a large number of photovoltaic cells and solar panels from abroad. Consequently, higher degrees of openness to trade can presumably lead to greater production of electricity from solar power. OIL refers to the per barrel real price of crude oil, measured in constant 2016 US dollar, and it accounts for the cross-price elasticity of REO in the country. A positive shock to world oil prices is likely to stimulate a transition from the use of the imported oils to renewable energy inputs for electricity generation purposes (Sadorsky, 2009). Finally, CO_2_ indicates the intensity of carbon emissions, measured in terms of kilograms per 2010 US dollars worth of real GDP. It is hypothesized that apprehensions regarding the atrocities of greenhouse gas emission-induced climate changes across the globe could trigger the urge to reduce the employment of the environmentally-unfriendly non-renewable energy resources to generate electricity and, therefore, facilitate RET (Crane et al., 2011).

Following Dogan and Inglesi-Lotz (2020), the STIRPAT modeling approach is modified in this study to assess the conditional impacts of FDI inflows on the ecological footprints of Bangladesh. The corresponding econometric model can be specified as:

2 $$\begin{aligned} \mathbf{lnEFP}_{\mathbf{t}} = & \varvec{~\beta }_{0} + \varvec{~\beta }_{1} \mathbf{lnFDI}_{\mathbf{t}} + \varvec{\beta }_{2} \mathbf{lnGDPPC}_{\mathbf{t}} + \varvec{\beta }_{3} \mathbf{lnGDPPC}_{\mathbf{t}}^{2} + \varvec{~\beta }_{4} \mathbf{lnREO}_{\mathbf{t}} + \varvec{\beta }_{5} \left( {\mathbf{lnREO}_{\mathbf{t}} \mathbf{*lnFDI}_{\mathbf{t}} } \right) \\ & + \varvec{~\beta }_{6} \mathbf{lnEI}_{\mathbf{t}} + \varvec{\beta }_{7} \mathbf{lnOPEN}_{\mathbf{t}} + \varvec{\beta }_{8} \mathbf{lnOIL}_{\mathbf{t}} + \varvec{\beta }_{9} \mathbf{lnURB}_{\mathbf{t}} + \mathbf{~\varepsilon }_{\mathbf{t}} \\ \end{aligned}$$

where the subscript t denotes the time period, β_i_ (i = 1, …, 9) are the elasticity parameters to be predicted. The variable EFP refers to the ecological footprints measured in terms of global hectares of land per capita. A rise in the total ecological footprints figures implicates environmental degradation while lower levels of ecological footprints indicate betterment of environmental quality. A positive sign of the elasticity parameter β_1_ would provide validity to the ‘*pollution haven hypothesis.*’ In contrast, a negative sign of the elasticity parameter β_1_ would authenticate the ‘*pollution halo hypothesis*’. The GDPPC figures and its squared term proxy for the impacts of affluence on the ecological footprints. The squared term is introduced into the model to assess the authenticity of the Environmental Kuznets Curve (EKC) hypothesis which postulates in favor of an inverted-U-shaped relationship between ecological footprints and national income levels (Li et al., 2021; Khan et al., 2021; Ahmad et al., 2021). In line with the principles of the EKC hypothesis, the signs of the elasticity parameters β_2_ and β_3_ can be expected to be positive and negative, respectively (Ulucak andBilgili, 2018). The sign of the elasticity parameter attached to FDI would implicate the impacts of FDI inflows on the ecological footprints. The statistical validity of the elasticity parameter attached to the interaction term between FDI inflows and REO would indicate a joint impact of these macroeconomic aggregates on the ecological footprints. The variable URB refers to the rate of urbanization in Bangladesh, measured in terms of the percentage share of the urban population in the total population of Bangladesh. The unplanned urbanization woes of Bangladesh are said to be the prime contributors to environmental degradation in Bangladesh (Ahmed & Islam, 2014). The statistical validity of the elasticity parameter attached to real crude oil prices, apart from reflecting the impacts of oil price shocks on the environment, would also indicate the strength of the cross-price elasticity of substitution between the oil consumption and renewable energy use (Murshed & Tanha, 2020). The sign of the elasticity parameter β_6_ can be expected to be negative provided there is the ease of substitution between oil and renewable energy resources and vice-versa.

Annual frequency data from 1972 to 2015 is used in this study. The variable REO is calculated from the electricity production data provided in the World Development Indicators (WDI) database of the World Bank. WDI provides data for Bangladesh's electricity production from non-renewable energy resources, namely oil, natural gas, and coal sources, as percentages of total electricity production in Bangladesh. Thus, the REO share is calculated by subtracting the share of the non-renewables from 100, which includes electricity generated from hydropower, solar and other sources. Data concerning all the other variables are sourced from WDI as well, except for the world crude oil price which is acquired from the British Petroleum Statistical Review of World Energy 2019 database. Data in the context of the ecological footprints are sourced from the Global Footprint Network (GFN, 2019) database. Table 6, in the appendix, summarizes the definitions and the data sources of the variables considered in this study. Moreover, the descriptive statistics and the correlation matrix are reported in Tables 7 and 8 ("see Appendix"). The variables lnFDI, lnGCF, lnCO2, lnOPEN, and lnEI are negatively skewed while the rest are positively skewed. Besides, all variables are platykurtic. The correlation matrix reveals strong correlations between the dependent and independent variables considered in this study. Furthermore, to check for multicollinearity issues in the models, the Variance Inflation Factor (VIF) analysis is conducted. The values of both the largest individual VIF and the mean VIF are below 10 which confirms that there are no multicollinearity issues.^1^

## Methodology

Before conducting the regression and causality exercises, it is pertinent to identify the stationarity and cointegrating properties of the variables. Thus, econometric analyses commence by applying appropriate unit root and cointegration techniques.

### Unit Root Analysis

Although most of the preceding empirical studies have suggested the application of the conventional unit root tests, including the Augmented Dickey-Fuller Dickey and Fuller (1979) and Phillips-Perron (1988) unit root testing methods, these methods generate biased estimates of the stationarity properties in the case of potential Structural Break (SB) issues in the data (Perron, 1989). Therefore, to counter the limitation of these techniques, this study uses the recently developed unit root analyses proposed by Narayan and Popp (2010). The Narayan-Popp (NP) method accounts for up to two SB in the data and generates unbiased test statistics to evaluate the order of integration among the variables included in the respective models. The NP technique predicts the stationarity properties using two model specifications. In one of the models, the two SB are assumed to be in the level while in the other model the two SB are assumed to be in the level and slope of a trending series (Narayan & Popp, 2010). For robustness check, the Clemente-Monantes-Reyes (CMR) unit root test (Clemenete et al., 1998), which also accounts for two SB in the data, is also applied. The test statistics under both the NP and CMR approaches are estimated under the null hypothesis on non-stationarity against the alternative hypothesis of stationarity; thus, the statistical significance of the test statistic confirms the stationarity of the corresponding variable. The unit root investigations are followed by the cointegration exercises.

### Cointegration Analysis

Cointegration exercises are performed to check the long-run associations between the variables included in the respective empirical model. The existence of long-run cointegrating relationships between the variables is a pre-requisite to estimating the long-run elasticities using appropriate regression techniques. This study primarily follows the Bayer-Hanck (BH) combined test for non-cointegration proposed by Bayer and Hanck (2013). The BH method prerequisites the variables to be commonly integrated in their respective first difference, I(1). This particular technique corrects for the limitations of the conventionally used Engle and Granger (1987) cointegration analysis due to being independent of certain nuisance parameters (Bayer & Hanck, 2013). Moreover, the BH approach provides efficient estimates of the cointegrating properties in the context of small samples. Besides, different cointegration tests are believed to provide dissimilar estimates of cointegration whereby generalizing the cointegration findings becomes a challenge. Thus, Bayer and Hanck (2013) came up with a solution to this problem by proposing a combined cointegration analysis which involves estimation of a Fisher statistic based on the following formula:

3 $${\mathbf{EG}} - {\mathbf{J}} \, = \, - {\mathbf{2}} \, \left[ {{\mathbf{ln}}\left( {{\mathbf{P}}_{{{\mathbf{EG}}}} } \right) \, + \, {\mathbf{ln}}\left( {{\mathbf{P}}_{{\mathbf{J}}} } \right)} \right]$$

4 $${\mathbf{EG}} - {\mathbf{J}} - {\mathbf{B}} - {\mathbf{BDM}} \, = \, - {\mathbf{2}} \, \left[ {{\mathbf{ln}}\left( {{\mathbf{P}}_{{{\mathbf{EG}}}} } \right) \, + \, {\mathbf{ln}}\left( {{\mathbf{P}}_{{\mathbf{J}}} } \right) \, + \, {\mathbf{ln}}\left( {{\mathbf{P}}_{{{\mathbf{BO}}}} } \right) \, + \, {\mathbf{ln}}\left( {{\mathbf{P}}_{{{\mathbf{BDM}}}} } \right)} \right]$$

where P refers to the probability values (*p* values) of individual cointegration tests; EG-J refers to the *p* values of the Fisher statistic for the combined Engle-Granger (EG) and Johansen (J) tests while EG-J-BO-BD refers to the *p* values of the Fisher statistic for the combined EG, J, Boswijik (1994) (B) and Banerjee, Dolado and Mestre (1998) (BDM) tests. The statistical significance of the Fisher statistic rejects the null hypothesis of no cointegration.

However, the BH method does not take into consideration the SB issues. Hence, this study also applies the Maki (2012) cointegration approach that can account for up to five SB in the data. There are four specific tests under the Maki cointegration approach: A (assumes the breaks in the intercept only), B (assumes the breaks in the intercepts and coefficients without the trend, C (assumes the break only in the intercept and coefficients but the model is assumed to have a trend) and D (assumes the breaks in the intercepts, coefficients and trend). The model specifications for these four tests can be shown as:

5 $$\rm Model\, A: y_{t} = \partial + \sum\nolimits_{{(i - = 1)}}^{m} {\partial _{i} D_{{(i,t)}} + \beta ^{{\prime }} x_{t} + u_{t} }$$

6 $$\rm Model\, B:{\mathbf{y}}_{{\mathbf{t}}} = \partial + \mathop \sum \limits_{{{\mathbf{i}} = 1}}^{{\mathbf{m}}} \partial_{{\mathbf{i}}} {\mathbf{D}}_{{{\mathbf{i}},{\mathbf{t}}}} + {\mathbf{\beta^{\prime}x}}_{{\mathbf{t}}} + \mathop \sum \limits_{{{\mathbf{i}} = 1}}^{{\mathbf{m}}} {{\varvec{\upbeta}}}_{{\mathbf{i}}}^{^{\prime}} {\mathbf{x}}_{{\mathbf{t}}} {\mathbf{D}}_{{{\mathbf{i}},{\mathbf{t}}}} + {\mathbf{u}}_{{\mathbf{t}}}$$

7 $$\rm Model\, C: {\mathbf{y}}_{{\mathbf{t}}} = \partial + \mathop \sum \limits_{{{\mathbf{i}} = 1}}^{{\mathbf{m}}} \partial_{{\mathbf{i}}} {\mathbf{D}}_{{{\mathbf{i}},{\mathbf{t}}}} + {\mathbf{\gamma t}} + {\mathbf{\beta^{\prime}x}}_{{\mathbf{t}}} + \mathop \sum \limits_{{{\mathbf{i}} = 1}}^{{\mathbf{m}}} {{\varvec{\upbeta}}}_{{\mathbf{i}}}^{^{\prime}} {\mathbf{x}}_{{\mathbf{t}}} {\mathbf{D}}_{{{\mathbf{i}},{\mathbf{t}}}} + {\mathbf{u}}_{{\mathbf{t}}}$$

8 $$\rm Model\, D: {\mathbf{y}}_{{\mathbf{t}}} = \partial + \mathop \sum \limits_{{{\mathbf{i}} = 1}}^{{\mathbf{m}}} \partial_{{\mathbf{i}}} {\mathbf{D}}_{{{\mathbf{i}},{\mathbf{t}}}} + {\mathbf{\gamma t}} + \mathop \sum \limits_{{{\mathbf{i}} = 1}}^{{\mathbf{m}}} {{\varvec{\upgamma}}}_{{\mathbf{i}}} {\mathbf{tD}}_{{{\mathbf{i}},{\mathbf{t}}}} + {\mathbf{\beta^{\prime}x}}_{{\mathbf{t}}} + \mathop \sum \limits_{{{\mathbf{i}} = 1}}^{{\mathbf{m}}} {{\varvec{\upbeta}}}_{{\mathbf{i}}}^{^{\prime}} {\mathbf{x}}_{{\mathbf{t}}} {\mathbf{D}}_{{{\mathbf{i}},{\mathbf{t}}}} + {\mathbf{u}}_{{\mathbf{t}}}$$

where y is the dependent variable and x is a vector of the independent variables. t denotes the time period, m denotes the highest number of breaks in the model which can take a maximum value of 5 and u denotes the error term. D_i,t_ is the dummy variable that is used to signal the presence of a break in the data. D_i,t_ takes a value of 1, denoting the existence of the SBs at specific break years T_bi_ (i = 1, …, m), if t_i_ are after the break years T_bi_ (i.e. t_i_ > T_bi_) and a value of 0, denoting no SB at the specific break years T_bi_ (i = 1, …, m), if t_i_ are before the break years T_bi_ (i.e. if t_i_ < T_bi_). Statistical significance of the test statistics in each of the four models (A, B, C, and D) rejects the null hypothesis of no cointegration to confirm the presence of cointegrating associations between the variables in the respective models. The identified SB from test A, within the Maki cointegration approach, is used to create SB dummy variables and included into the respective empirical models to control for time fixed effects. The regression analysis follows the cointegration exercises.

### The Autoregressive Distributed Lag-Error Correction Model Approach

Following Hassan et al. (2019), the short and long-run elasticities are estimated using the Autoregressive Distributed Lag (ARDL) technique introduced by Pesaran et al. (2001). This regression model is chosen in the context of this study due to several of its beneficial features: (a) it can accommodate a mixed order of integration among the variables included in the respective model and generates unbiased elasticity estimates compared to the other estimation techniques that pre-requisite a common order of integration among the variables; (b) it can predict both the short- and long-run elasticities unlike the other conventionally used estimators which primarily focuses on the long-run effects only; (c) this technique is efficient in accounting for the possibility of endogeneity and serial correlation problems in the data; and (d) it is the most efficient estimator for handling data sets with short time dimensions. However, a limitation of this procedure is its inability to accommodate the SB in the data. Thus, the inclusion of the SB year dummies into the econometric models helps to resolve this problem. The ARDL approach is a two-step procedure. Firstly, it involves the estimation of an unrestricted Error-Correction Model (ECM). The ECM model in the context of model (1) can be specified as:

9 $$\begin{aligned} \Delta {\mathbf{lnREO}}_{{\mathbf{t}}} \,=\, & {{\varvec{\upbeta}}}_{0} + {{\varvec{\upbeta}}}_{1} {\mathbf{lnREO}}_{{{\mathbf{t}} - 1}} + {{\varvec{\upbeta}}}_{2} {\mathbf{lnFDI}}_{{{\mathbf{t}} - 1}} + {{\varvec{\upbeta}}}_{3} {\mathbf{lnEI}}_{{{\mathbf{t}} - 1}} + {{\varvec{\upbeta}}}_{4} {\mathbf{lnGDPPC}}_{{{\mathbf{t}} - 1}} + {{\varvec{\upbeta}}}_{5} {\mathbf{lnGCF}}_{{{\mathbf{t}} - 1}} \\ & + {{\varvec{\upbeta}}}_{6} {\mathbf{lnOPEN}}_{{{\mathbf{t}} - 1}} + {{\varvec{\upbeta}}}_{7} {\mathbf{lnOIL}}_{{{\mathbf{t}} - 1}} + {{\varvec{\upbeta}}}_{8} {\mathbf{lnCO}}2_{{{\mathbf{t}} - 1}} \\ & + {{\varvec{\upbeta}}}_{9} {\mathbf{D}}_{{{\mathbf{t}} - 1}} + \mathop \sum \limits_{{{\mathbf{i}} = 1}}^{{\mathbf{q}}} {{\varvec{\upalpha}}}_{{1{\mathbf{i}}}} \Delta {\mathbf{lnREO}}_{{{\mathbf{t}} - {\mathbf{i}}}} + \mathop \sum \limits_{{{\mathbf{j}} = 0}}^{{\mathbf{r}}} {{\varvec{\upalpha}}}_{{2{\mathbf{j}}}} \Delta {\mathbf{lnFDI}}_{{{\mathbf{t}} - {\mathbf{j}}}} + \mathop \sum \limits_{{{\mathbf{k}} = 0}}^{{\mathbf{s}}} {{\varvec{\upalpha}}}_{{3{\mathbf{k}}}} \Delta {\mathbf{lnEI}}_{{{\mathbf{t}} - {\mathbf{k}}}} \\ & + \mathop \sum \limits_{{{\mathbf{l}} = 0}}^{{\mathbf{t}}} {{\varvec{\upalpha}}}_{{4{\mathbf{l}}}} \Delta {\mathbf{lnGDPPC}}_{{{\mathbf{t}} - {\mathbf{l}}}} + \mathop \sum \limits_{{{\mathbf{m}} = 0}}^{{\mathbf{u}}} {{\varvec{\upalpha}}}_{{5{\mathbf{m}}}} \Delta {\mathbf{lnGCF}}_{{{\mathbf{t}} - {\mathbf{m}}}} + \mathop \sum \limits_{{{\mathbf{n}} = 0}}^{{\mathbf{v}}} {{\varvec{\upalpha}}}_{{6{\mathbf{n}}}} \Delta {\mathbf{lnOPEN}}_{{{\mathbf{t}} - {\mathbf{n}}}} \\ & + \mathop \sum \limits_{{{\mathbf{o}} = 0}}^{{\mathbf{w}}} {{\varvec{\upalpha}}}_{{7{\mathbf{o}}}} \Delta {\mathbf{lnOIL}}_{{{\mathbf{t}} - {\mathbf{o}}}} + \mathop \sum \limits_{{{\mathbf{p}} = 0}}^{{\mathbf{x}}} {{\varvec{\upalpha}}}_{{8{\mathbf{p}}}} \Delta {\mathbf{lnCO}}2_{{{\mathbf{t}} - {\mathbf{p}}}} + \mathop \sum \limits_{{{\mathbf{z}} = 0}}^{{\mathbf{y}}} {{\varvec{\upalpha}}}_{{9{\mathbf{z}}}} \Delta {\mathbf{D}}_{{{\mathbf{t}} - {\mathbf{z}}}} + {{\varvec{\uptheta}}}_{{\mathbf{t}}} \\ \end{aligned}$$

where ∆ denotes first difference form of the corresponding variables. The short run dynamics for model (1) is observed from the model below:

10 $$\begin{aligned} \Delta {\mathbf{lnREO}}_{{\mathbf{t}}} \,=\, & {{\varvec{\upgamma}}}_{1} + \mathop \sum \limits_{{{\mathbf{i}} = 1}}^{{\mathbf{m}}} {{\varvec{\upgamma}}}_{{1{\mathbf{i}}}} \Delta {\mathbf{lnREO}}_{{{\mathbf{t}} - {\mathbf{i}}}} + \mathop \sum \limits_{{{\mathbf{j}} = 0}}^{{\mathbf{n}}} {{\varvec{\upgamma}}}_{{2{\mathbf{j}}}} \Delta {\mathbf{lnFDI}}_{{{\mathbf{t}} - {\mathbf{j}}}} \\ & + \mathop \sum \limits_{{{\mathbf{k}} = 0}}^{{\mathbf{o}}} {{\varvec{\upgamma}}}_{{3{\mathbf{k}}}} \Delta {\mathbf{lnEI}}_{{{\mathbf{t}} - {\mathbf{k}}}} + \mathop \sum \limits_{{{\mathbf{l}} = 0}}^{{\mathbf{p}}} {{\varvec{\upgamma}}}_{{4{\mathbf{l}}}} \Delta {\mathbf{lnGDPPC}}_{{{\mathbf{t}} - {\mathbf{l}}}} \\ & + \mathop \sum \limits_{{{\mathbf{s}} = 0}}^{{\mathbf{q}}} {{\varvec{\upgamma}}}_{{5{\mathbf{s}}}} \Delta {\mathbf{lnGCF}}_{{{\mathbf{t}} - {\mathbf{s}}}} + \mathop \sum \limits_{{{\mathbf{v}} = 0}}^{{\mathbf{r}}} {{\varvec{\upgamma}}}_{{6{\mathbf{v}}}} \Delta {\mathbf{lnOPEN}}_{{{\mathbf{t}} - {\mathbf{v}}}} + \mathop \sum \limits_{{{\mathbf{w}} = 0}}^{{\mathbf{a}}} {{\varvec{\upgamma}}}_{{7{\mathbf{w}}}} \Delta {\mathbf{lnOIL}}_{{{\mathbf{t}} - {\mathbf{w}}}} \\ & + \mathop \sum \limits_{{{\mathbf{x}} = 0}}^{{\mathbf{b}}} {{\varvec{\upgamma}}}_{{8{\mathbf{x}}}} \Delta {\mathbf{CO}}2_{{{\mathbf{t}} - {\mathbf{x}}}} + \mathop \sum \limits_{{{\mathbf{y}} = 0}}^{{\mathbf{c}}} {{\varvec{\upgamma}}}_{{9{\mathbf{y}}}} \Delta {\mathbf{D}}_{{{\mathbf{t}} - {\mathbf{y}}}} + {\mathbf{\pi ECT}}_{{{\mathbf{t}} - 1}} + {{\varvec{\uptau}}}_{{\mathbf{t}}} \\ \end{aligned}$$

where ECT_t-1_ represents the one-period lagged Error-Correction Term (ECT) which is estimated from the residuals of the long-run equation shown in Eq. (11). The ECT reflects the speed of adjustment of the variables in restoring long-run equilibrium following a deviation in the earlier period. The long-run elasticities are sourced from the model below:

11 $$\begin{aligned} {\mathbf{lnREO}}_{{\mathbf{t}}} \,=\, & \partial_{0} + \partial_{1} {\mathbf{lnREO}}_{{{\mathbf{t}} - 1}} + \partial_{2} {\mathbf{lnEI}}_{{{\mathbf{t}} - 1}} + \partial_{3} {\mathbf{lnFDI}}_{{{\mathbf{t}} - 1}} + \partial_{4} {\mathbf{lnGDPPC}}_{{{\mathbf{t}} - 1}} \\ & + \partial_{5} {\mathbf{lnGCF}}_{{{\mathbf{t}} - 1}} + \partial_{6} {\mathbf{lnOPEN}}_{{{\mathbf{t}} - 1}} + \partial_{7} {\mathbf{lnOIL}}_{{{\mathbf{t}} - 1}} + \partial_{8} {\mathbf{lnCO}}2_{{{\mathbf{t}} - 1}} + \partial_{9} {\mathbf{D}}_{{{\mathbf{t}} - 1}} + \vartheta_{{\mathbf{t}}} \\ \end{aligned}$$

Similarly, in the context of model (2) the ARDL model can be expressed as:

12 $$\begin{aligned} \Delta {\mathbf{lnEFP}}_{{\mathbf{t}}} \,=\, & ~{\mathbf{\beta }}_{0} + ~{\mathbf{\beta }}_{1} {\mathbf{lnEFP}}_{{{\mathbf{t}} - 1}} + {\mathbf{\beta }}_{2} {\mathbf{lnFDI}}_{{{\mathbf{t}} - 1}} + {\mathbf{\beta }}_{3} {\mathbf{lnGDPPC}}_{{{\mathbf{t}} - 1}} + {\mathbf{\beta }}_{4} {\mathbf{lnGDPPC}}^{2} _{{{\mathbf{t}} - 1}} + ~{\mathbf{\beta }}_{5} {\mathbf{lnREO}}_{{{\mathbf{t}} - 1}} + {\mathbf{\beta }}_{6} \left( {{\mathbf{lnREO}}*{\mathbf{lnFDI}}} \right)_{{{\mathbf{t}} - 1}} + {\mathbf{\beta }}_{7} {\mathbf{lnEI}}_{{{\mathbf{t}} - 1}} + {\mathbf{\beta }}_{8} {\mathbf{lnOPEN}}_{{{\mathbf{t}} - 1}} \\ & + {\mathbf{\beta }}_{9} {\mathbf{lnOIL}}_{{{\mathbf{t}} - 1}} + {\mathbf{\beta }}_{{10}} {\mathbf{lnURB}}_{{{\mathbf{t}} - 1}} + {\mathbf{\beta }}_{{11}} {\mathbf{D}}_{{{\mathbf{t}} - 1}} + ~\mathop \sum \limits_{{{\mathbf{i}} = 1}}^{{\mathbf{q}}} {\mathbf{\alpha }}_{{1{\mathbf{i}}}} \Delta {\mathbf{lnEFP}}_{{{\mathbf{t}} - {\mathbf{i}}}} \\ & + ~\mathop \sum \limits_{{{\mathbf{j}} = 0}}^{{\mathbf{r}}} {\mathbf{\alpha }}_{{2{\mathbf{j}}}} \Delta {\mathbf{lnFDI}}_{{{\mathbf{t}} - {\mathbf{j}}}} + ~\mathop \sum \limits_{{{\mathbf{k}} = 0}}^{{\mathbf{s}}} {\mathbf{\alpha }}_{{3{\mathbf{k}}}} \Delta {\mathbf{lnGDPPC}}_{{{\mathbf{t}} - {\mathbf{k}}}} + ~\mathop \sum \limits_{{{\mathbf{l}} = 0}}^{{\mathbf{t}}} {\mathbf{\alpha }}_{{4{\mathbf{l}}}} \Delta {\mathbf{lnGDPPC}}^{2} _{{{\mathbf{t}} - {\mathbf{l}}}} \\ & + \mathop \sum \limits_{{{\mathbf{m}} = 0}}^{{\mathbf{u}}} {\mathbf{\alpha }}_{{5{\mathbf{m}}}} \Delta {\mathbf{lnREO}}_{{{\mathbf{t}} - {\mathbf{m}}}} + \mathop \sum \limits_{{{\mathbf{n}} = 0}}^{{\mathbf{v}}} {\mathbf{\alpha }}_{{6{\mathbf{n}}}} \Delta \left( {{\mathbf{lnREO}}*{\mathbf{lnFDI}}} \right)_{{{\mathbf{t}} - {\mathbf{n}}}} + ~\mathop \sum \limits_{{{\mathbf{o}} = 0}}^{{\mathbf{w}}} {\mathbf{\alpha }}_{{7{\mathbf{o}}}} \Delta {\mathbf{lnEI}}_{{{\mathbf{t}} - {\mathbf{o}}}} \\ & + ~\mathop \sum \limits_{{{\mathbf{p}} = 0}}^{{\mathbf{x}}} {\mathbf{\alpha }}_{{8{\mathbf{p}}}} \Delta {\mathbf{lnOPEN}}_{{{\mathbf{t}} - {\mathbf{p}}}} + ~\mathop \sum \limits_{{{\mathbf{q}} = 0}}^{\user2{y}} {\mathbf{\alpha }}_{{9{\mathbf{q}}}} \Delta {\mathbf{lnOIL}}_{{{\mathbf{t}} - {\mathbf{q}}}} + ~\mathop \sum \limits_{{{\mathbf{r}} = 0}}^{\user2{z}} {\mathbf{\alpha }}_{{10\user2{r}}} \Delta {\mathbf{lnURB}}_{{{\mathbf{t}} - {\mathbf{r}}}} + \mathop \sum \limits_{{{\mathbf{s}} = 0}}^{\user2{a}} {\mathbf{\alpha }}_{{11{\mathbf{s}}}} \Delta {\mathbf{D}}_{{{\mathbf{t}} - {\mathbf{s}}}} + {\mathbf{\theta }}_{{\mathbf{t}}} ~ \\ \end{aligned}$$

The short run dynamics for model (2) is observed from the model below:

13 $$\begin{aligned} \Delta {\mathbf{lnEFP}}_{{\mathbf{t}}} \,=\, & {{\varvec{\upgamma}}}_{1} + \mathop \sum \limits_{{{\mathbf{i}} = 1}}^{{\mathbf{m}}} {{\varvec{\upgamma}}}_{{1{\mathbf{i}}}} \Delta {\mathbf{lnEFP}}_{{{\mathbf{t}} - {\mathbf{i}}}} + \mathop \sum \limits_{{{\mathbf{j}} = 0}}^{{\mathbf{n}}} {{\varvec{\upgamma}}}_{{2{\mathbf{j}}}} \Delta {\mathbf{lnFDI}}_{{{\mathbf{t}} - {\mathbf{j}}}} + \mathop \sum \limits_{{{\mathbf{k}} = 0}}^{{\mathbf{o}}} {{\varvec{\upgamma}}}_{{3{\mathbf{k}}}} \Delta {\mathbf{lnGDPPC}}_{{{\mathbf{t}} - {\mathbf{k}}}} + \mathop \sum \limits_{{{\mathbf{l}} = 0}}^{{\mathbf{p}}} {{\varvec{\upgamma}}}_{{4{\mathbf{l}}}} \Delta {\mathbf{lnGDPPC}}^{2}_{{{\mathbf{t}} - {\mathbf{l}}}} \\ & + \mathop \sum \limits_{{{\mathbf{s}} = 0}}^{{\mathbf{q}}} {{\varvec{\upgamma}}}_{{5{\mathbf{s}}}} \Delta {\mathbf{lnREO}}_{{{\mathbf{t}} - {\mathbf{s}}}} + \mathop \sum \limits_{{{\mathbf{v}} = 0}}^{{\mathbf{r}}} {{\varvec{\upgamma}}}_{{6{\mathbf{v}}}} \left( {\Delta {\mathbf{lnREO}}*{\mathbf{lnFDI}}} \right)_{{{\mathbf{t}} - {\mathbf{v}}}} + \\ & + \mathop \sum \limits_{{{\mathbf{w}} = 0}}^{{\mathbf{a}}} {{\varvec{\upgamma}}}_{{7{\mathbf{w}}}} \Delta {\mathbf{lnEI}}_{{{\mathbf{t}} - {\mathbf{w}}}} + \mathop \sum \limits_{{{\mathbf{x}} = 0}}^{{\mathbf{b}}} {{\varvec{\upgamma}}}_{{8{\mathbf{x}}}} \Delta {\mathbf{lnOPEN}}_{{{\mathbf{t}} - {\mathbf{x}}}} + + \mathop \sum \limits_{{{\mathbf{y}} = 0}}^{{\varvec{c}}} {{\varvec{\upgamma}}}_{{9{\varvec{y}}}} \Delta {\mathbf{lnOIL}}_{{{\mathbf{t}} - {\mathbf{y}}}} \\ & + \mathop \sum \limits_{{{\mathbf{z}} = 0}}^{{\varvec{d}}} {{\varvec{\upgamma}}}_{{10{\varvec{z}}}} \Delta {\mathbf{lnURB}}_{{{\mathbf{t}} - {\mathbf{z}}}} + \mathop \sum \limits_{{{\mathbf{l}} = 0}}^{{\varvec{e}}} {{\varvec{\upgamma}}}_{{11{\varvec{l}}}} \Delta {\mathbf{D}}_{{{\mathbf{t}} - {\mathbf{i}}}} + {\mathbf{\pi ECT}}_{{{\mathbf{t}} - 1}} + {{\varvec{\uptau}}}_{{\mathbf{t}}} \\ \end{aligned}$$

The long-run elasticities for model (2) are sourced from the model below:

14 $$\begin{aligned} {\mathbf{lnEFP}}_{{\mathbf{t}}} \,=\, & \partial_{0} + \partial_{1} {\mathbf{lnEFP}}_{{{\mathbf{t}} - 1}} + \partial_{2} {\mathbf{lnFDI}}_{{{\mathbf{t}} - 1}} + \partial_{3} {\mathbf{lnGDPPC}}_{{{\mathbf{t}} - 1}} + \partial_{4} {\mathbf{lnGDPPC}}^{2}_{{{\mathbf{t}} - 1}} + \partial_{5} {\mathbf{lnREO}}_{{{\mathbf{t}} - 1}} \\ & + (\partial_{6} {\mathbf{lnREO}}*{\mathbf{lnFDI}})_{{{\mathbf{t}} - 1}} + \partial_{7} {\mathbf{lnEI}}_{{{\mathbf{t}} - 1}} + \partial_{8} {\mathbf{lnOPEN}}_{{{\mathbf{t}} - 1}} \\ & + \partial_{9} {\mathbf{lnOIL}}_{{{\mathbf{t}} - 1}} + \partial_{10} {\mathbf{lnURB}}_{{{\mathbf{t}} - 1}} + \partial_{11} {\mathbf{D}}_{{{\mathbf{t}} - 1}} + \vartheta_{{\mathbf{t}}} \\ \end{aligned}$$

The stability of the ARDL estimates are checked using the Breusch-Godfrey Lagrange Multiplier test for Autocorrelation (BGodfrey) proposed by Breusch and Godfrey (1981), the Lagrange Multiplier test for the presence of Autoregressive Conditional Heteroskedasticity (ARCH) proposed by Engle (1982), and the Cumulative Sum (CUSUM) and Cumulative Sum of Squares (CUSUMSQ) methods proposed by Chow (1960) and Brown et al. (1975), respectively.

### The Hacker and Hatemi-J Causality Analysis

The causal associations between the variables are evaluated using the Hacker and Hatemi-J (HH) causality estimation technique proposed by Hacker and Hatemi-J (2012). This method is modified from the bootstrapped causality test proposed by Hacker and Hatemi-J (2006). According to Hacker and Hatemi-J (2006), bootstrapping the distribution reduces the concerns from small sample size distortions associated with the conventional Wald test introduced by Toda and Yamamoto (TY) (1995), irrespective of the presence of autoregressive conditional heteroscedasticity effects within the model. The modification of the modified Wald test statistic of Hacker and Hatemi-J (2006) is done by endogenizing the optimal lag selection criterion which tends to reduce the small sample size distortions further. The HH technique uses a Vector Autoregressive (VAR) model to calculate the modified Wald test statistics under the null hypothesis of no causality between the dependent and the independent variables against the alternative hypothesis of otherwise. The bootstrapping involves two stages: firstly, estimating the optimal lag structure and secondly predicting the Wald statistic for investing the Granger causality. The VAR model of order k can be specified as:

15 $${\mathbf{y}}_{{\mathbf{t}}} = {{\varvec{\upbeta}}}_{0} + {{\varvec{\upbeta}}}_{1} {\mathbf{y}}_{{{\mathbf{t}} - 1}} + \cdots + {{\varvec{\upbeta}}}_{{\mathbf{k}}} {\mathbf{y}}_{{{\mathbf{t}} - {\mathbf{k}}}} + {\mathbf{u}}_{{\mathbf{t}}}$$

where y_t_, β_0_ and u_t_ are vectors with dimension n × 1 and B_i_ (i > 0) is a parameter matric with a dimension of n × n. The error term u_t_ has no expected value and presumed to be independent and identically distributed with a non-singular covariance matrix (Hacker & Hatemi-J, 2012). In the first stage of the bootstrapping approach under the HH approach, Eq. (12) is estimated without imposing any restriction in terms of the non-causality null hypothesis. The predicted value, y*, can be given by:

16 $${\mathbf{y}}^{*}_{{\mathbf{t}}} = {\hat{\mathbf{\beta }}}_{0} + {\hat{\mathbf{\beta }}}_{1} {\mathbf{y}}_{{{\mathbf{t}} - 1}} + \cdots + {\hat{\mathbf{\beta }}}_{{\mathbf{k}}} {\mathbf{y}}_{{{\mathbf{t}} - {\mathbf{k}}}} + \widehat{{{\mathbf{u}}*}}_{{\mathbf{t}}}$$

where $\widehat{{{\mathbf{u}}*}}_{{\mathbf{t}}}$ is a vector of bootstrapped error terms and t (t = 1, …, T) is the time period. The set of T bootstrapped error term vectors is estimated by drawing randomly with replacement from the vectors of the modified residual to ensure that the mean value of the bootstrapped error term vectors is a zero vector. The modified residuals are the raw residuals modified via leverages within the HH approach. This modification is ideal to deal with heteroscedasticity issues within the model and also to account for the ARCH effects (Hacker and Hatemi, 2012). The bootstrapping mechanism is repeated M times to produce a Wald statistic each time which is based on the TY methodology. The resulting set of bootstrapped Wald statistics is termed as the bootstrapped Wald distribution which is then used to evaluate the causal properties of the pair of variables. In the case of the predicted Wald –statistic being greater than the corresponding bootstrapped critical value then causality stemming from the independent to the dependent variable is affirmed via rejection of the null hypothesis of non-causality. For comparison purposes, the TY causality technique is also applied.

## Results and Discussions

This section begins by analyzing the findings from the unit root and cointegration analyses followed by the discussion on the regression and causality outcomes.

### Unit Root Results

The NP and CMR unit root test results are reported in Table 1. The results reveal that all the variables are non-stationary at their respective level forms but they become stationary at their first differences, irrespective of considering the SB in level or both level and slope. Besides, the identical results from the CMR unit root analysis confirm the robustness of the stationarity properties of the variables. Hence, the unit root results implicate a common order of integration among the variables, at first difference. Therefore, all the variables included in the empirical analyses are mean-reverting which nullifies the possibility of the regression analysis to follow being spurious.

**Table 1:** The Narayan and Popp (2010) and Clemente-Monantes-Reyes (1998) unit root test results

Variable	Level, I(0)				1st Difference, I(1)				Level, I(0)				1st Difference, I(1)			
	Test stat	k	BY1	BY2	Test stat	k	BY1	BY2	Test stat	k	BY1	BY2	Test stat	k	BY1	BY2
Narayan and Popp (2010) unit root test																
Considering two structural breaks in the level									Considering two structural breaks in the level and slope							
lnREO	− 2.886	3	1999	2002	− 6.199***	2	2000	2003	− 3.428	3	1999	2002	− 9.433***	2	2000	2003
lnFDI	2.210	4	1996	2001	6.643***	2	1999	2001	− 2.086	3	1996	2001	5.811**	2	1999	2001
lnGDPPC	− 2.704	2	1998	2007	− 5.581***	4	1998	2007	− 2.814	2	1994	2008	− 6.281***	3	1998	2006
lnGCF	− 0.548	3	2002	2013	5.818***	3	2004	2013	− 0.748	3	2002	2013	− 5.902**	2	2004	2010
lnOPEN	− 3.004	2	1996	2004	− 7.079***	3	1994	2005	− 3.119	3	1999	2009	− 8.128***	3	1995	2005
lnOIL	− 2.701	2	1998	2004	− 5.914***	2	2000	2005	− 2.225	1	1998	2005	− 5.993**	2	2002	2007
lnCO_2_	− 3.519	3	1995	2000	− 8.482***	3	1997	2000	− 2.851	2	1995	2002	− 9.090***	2	1997	2000
lnEFP	− 1.998	2	2001	2009	− 7.221***	2	2000	2007	− 2.519	2	2000	2010	− 7.902***	2	2002	2010
lnURB	− 1.892	3	2002	2007	− 5.999***	3	2001	2010	− 1.221	3	2000	2007	− 6.001***	3	2003	2010
lnEI	− 2.790	2	1996	2007	− 4.818**	2	2002	2006	− 2.459	2	1999	2008	− 7.212***	2	2002	2009

Variable	Level, I(0)				1st Difference, I(1)			
	Test stat	k	BY1	BY2	Test stat	K	BY1	BY2
Clemente-Monantes-Reyes (1998) unit root test								
lnREO	0.523	3	1999	2003	− 5.051***	2	2001	2006
lnFDI	1.091	4	1996	2001	6.242***	2	1997	2004
lnGDPPC	1.037	2	2000	2008	7.139***	4	1994	2005
lnGCF	0.147	3	2001	2011	7.816***	3	2002	2009
lnOPEN	0.625	2	1998	2002	6.702***	3	1994	2006
lnOIL	1.118	2	1999	2005	3.005***	2	2001	2006
lnCO_2_	− 0.334	3	1995	2000	2.730***	3	1996	2001
lnEFP	0.206	2	2000	2007	4.394***	2	2000	2009
lnURB	0.225	3	2005	2006	5.024***	3	2003	2010
lnEI	0.680	2	1999	2007	− 6.039***	2	2000	2010

The test statistics are estimated under the null hypothesis on non-stationarity against the alternative hypothesis of stationarity; k denotes the optimal lags based on Akaike Information Criterion (AIC), are provided within the parentheses; BY1 and BY2 refer to the locations of the first and second break years in the data; the asterisks *** and ** denote statistical significance at 10% and 5% significance levels, respectively

### Cointegration Results

Table 2 presents the results from cointegration analysis. In the context of the BH test, the statistical significance of the EG-J-BO-BDM test statistics, at 1% level, of the estimated test statistics confirms cointegrating associations between the variables of concern in the respective models. Besides, the statistical significance, at 1% and 5% levels, of the test statistics under the Maki (2012) approach also affirm the cointegrating relationships; thus, the results can be considered robust across different estimation techniques. In line with these results, it can be held that FDI inflows, REO shares, ecological footprints and the relevant control variables have long-run associations. These findings fulfill the pre-requisite to predicting the long-run elasticities. Moreover, the results from the Maki cointegration analysis also spit out the locations of five structural break points for each of the two empirical models. Accordingly, the identified break points, from test A under the Maki cointegration approach, are used to create break year dummies for inclusion into the respective models in order to accommodate these data issues within the regression analysis.

**Table 2:** The Bayer-Hanck (2013) and Maki (2012) cointegration test results

Model (1)		Model (2)	
Bayer and Hanck (2013) cointegration			
EG-JOH stat	EG-J-BO-BDM stat	EG-JOH stat	EG-J-BO-BDM stat
7.162	18.246***	7.778	19.113***

	Model (1)		Model (2)	
Maki (2012) cointegration analysis				
Test	Test Stat	BY	Test Stat	BY
A	− 7.89***	1995, 1997, 1999, 2004, 2010	− 7.99***	1978, 1982, 1991, 1998, 2007
B	− 7.01**	1995, 1998, 2001, 2005, 2009	− 7.29***	1980, 1986, 1991, 1999, 2014
C	− 18.01***	1989, 1995, 1999, 2001, 2009	− 18.98***	1978, 1983, 1995, 1999, 2011
D	− 9.05	1980, 1985, 2005, 2009, 2013	− 9.02	1980, 1988, 1999, 2001, 2010

The test statistics are estimated under the null hypothesis on no cointegration against the alternative hypothesis of cointegration; The optimal lags selection are based on AIC; BY denotes the five Break Years;*** and ** denote statistical significance at 1% and 5% significance levels

### Regression Results

The ARDL short- and long-run elasticity estimates in the context of model (1) are reported in Table 3. The short-run elasticity estimates, accounting for the structural breaks in the data, reveal that incoming FDIs in Bangladesh exert a dampening impact on the nation’s REO shares. This can be perceived from the negative signs and statistical significance of the predicted elasticity parameters attached to current and lagged levels of FDI inflows. Hence, incoming FDI is found to be ineffective in facilitating the RET phenomenon in Bangladesh in the short-run. However, it is to be noted that the adverse impacts of incoming FDI on the REO shares tend to decline with time since the elasticity estimate at the current level of FDI inflows is relatively smaller than that of the corresponding elasticities at the lagged forms of FDI inflows. These imply that although FDI inflows initially depress the REO shares, it goes on to improve the quality of the environment as the volume of FDI inflows tend to persistently go up. Hence, it can be stated that the quality of the FDI flowing into Bangladesh possibly improves with time whereby the marginal negative impacts on the REO shares tend to diminish. Besides, a particular reason behind incoming FDI undermining the shares of renewable electricity in the aggregate electricity output figures of Bangladesh could be because of the fact that the FDI flowing into the country are predominantly directed at industries that are relatively more intensive in the use of non-renewable electricity; thus, marginalizing the overall REO shares. These short-run findings are parallel to the negative FDI inflow-REO nexus found by Lin and Li (2015) for China while contradicting the assertions made by Lin et al. (2016) where the authors failed to establish any statistically significant relationship between these variables in the case of China. Similarly, Kilicarslan (2019) also opined in favor of FDI inflows in the short-run being unable to explain the variations in the REO figures in Brazil, Russia, India, China South Africa, and Turkey.

**Table 3:** The short and long-run elasticity estimates from the ARDL approach for model (1)

Dep. variable: lnREO			
Short-run analysis		Long-run analysis	
∆(REO (− 1))	0.115 (0.809)	lnFDI	0.053*** (0.009)
∆(lnFDI)	− 0.109** (0.505)	lnEI	− 0.824** (0.413)
∆(lnFDI (− 1))	− 0.689** (0.345)	lnGDPPC	− 0.154** (0.075)
∆(lnFDI (− 2))	− 1.115*** (0.433)	lnGCF	− 3.683 (2.921)
∆(lnEI)	− 0.212*** (0.082)	lnOPEN	− 1.575*** (0.423)
∆(lnGDPPC)	− 2.145** (2.071)	lnOIL	4.299*** (2.412)
∆(lnGDPPC (− 1))	− 4.793*** (1.911)	lnCO_2_	− 0.485*** (0.199)
∆(GCF)	− 4.283*** (1.892)	BY1	0.260*** (0.040)
∆(GCF (− 1))	− 4.185** (2.090)	BY2	0.401** (0.199)
∆(lnOPEN)	− 0.923** (0.451)	BY3	− 0.090*** (0.0210
∆(lnOIL)	3.092*** (1.001)	BY4	− 1.330*** (0.400)
∆(lnOIL (− 1))	2.598** (1.254)	BY5	0.190 (0.121)
∆(lnOIL (− 2))	0.118** (0.061)	Adj. R2	0.769
∆(lnCO_2_))	− 0.851 (0.640)	Obvs	45
∆(lnCO_2_ (− 1))	− 1.330 (0.925)	Diagnostics	
∆(BY1)	0.225** (0.112)	BGodfrey	0.329
∆(BY2)	0.349*** (0.101)	ARCH	1.156
∆(BY3)	− 0.122** (0.061)	CUSUM	Stable
∆(BY4)	− 1.209*** (0.340)	CUSUMSQ	Stable
∆(BY5)	0.223** (0.112)		
ECT_t-1_	− 0.781*** (0.232)		

The standard errors are reported within the parentheses; Optimal lag selection is based on the AIC; *** and ** denote statistical significance at 1% and 5% significance levels

Among the other short-run determinants of REO in Bangladesh, technological advancement is found to increase the REO shares. This is evident from the statistical significance of the negative elasticity parameter attached to the current level of energy use intensity. This negative correlation implies that as the intensity of energy use decreases, which is synonymous with technological advancement, the REO shares are likely to go up. Hence, it can be said that technological advancement is indeed a key determinant of higher REO in Bangladesh. A 1% decline in the energy intensity level is predicted to elevate the REO shares in the short-run by 0.21%, on average, *ceteris paribus.* Murshed (2021d) also found similar results and quoted that technological innovation-led energy efficiency improvements govern renewable electricity transition across South Asia.

Besides, economic growth is found to favor the use of electricity generated from the non-renewable energy resources, thus, reducing the REO shares. However, much like the case in the context of FDI inflows, economic growth is also evidenced to lower the REO shares. A percentage change in the one-period lagged level of the real per capita GDP of Bangladesh reduces the REO shares by 4.73% whereas a percentage change in the current level of real per capita GDP reduces the REO shares by 2.135%, on average, *ceteris paribus.* Hence, it can be asserted that higher levels of economic growth tend to empower the Bangladesh economy to gradually overcome the constraints that inhibit renewable electricity production. A similar negative, but statistically insignificant, short-run correlation between per capita real GDP and aggregate renewable energy use was reported by Fan and Hao (2020) in the context of 31 Chinese provinces. Likewise economic growth, the short-run REO-inhibiting impacts of domestic capital investments in the Bangladesh economy are witnessed. A percentage change in the current and one-period lagged levels of gross fixed capital formations in Bangladesh is found to depress the REO shares by 4.28% and 4.19%, on average, *ceteris paribus.* Furthermore, involvement in international trade is also found to suppress the REO shares which can be rationalized by the claim that the export sector of Bangladesh is predominantly dependent on the use of non-renewable electric power. Bangladesh relies heavily on its ready-made garments exports due to pursuing an export-led growth strategy (Shafiullah and Navatnam, 2016). However, the associated industries overwhelmingly intensive in the use of non-renewable electricity (Paul et al., 2017) whereby higher openness to trade justifiably dampens the REO shares. The short-run elasticity in this regard shows that a percentage increase in the trade openness index is associated with a decline in the REO shares by 0.92%, on average, *ceteris paribus.* This finding opposes the statistically insignificant short-run trade openness-REO nexus found by Khraief et al. (2018) in the context of Algeria.

In contrast, exogenous positive shocks to real crude oil prices in the international oil markets are predicted to stimulate a substitution effect that can plausibly be linked to lower use of imported oils for electricity generation purposes in Bangladesh. The positive signs of the statistically significant elasticity parameters attached to the current and lagged forms of the real crude oil price variable affirm this claim. Notably, the magnitude of the elasticity parameter attached to the current level of crude oil price is relatively higher than that attached to the lagged levels of crude oil prices. These imply that transitioning from the use of non-renewable to renewable resources for electricity generation is relatively difficult as the real oil price starts to rise. However, persistent rises in the oil prices with time gradually reduces the oil-dependencies which, in turn, can also be linked to increments in the REO shares, simultaneously. The initial detrimental impacts of such imported oil-dependency on the REO shares were also reported by Murshed and Tanha (2020) for four South Asian net oil-importing nations. The finding of the negative correlation between oil price and REO is in line with the findings put forward by Shahzad et al. (2021) from the understanding that the authors claimed that higher crude oil prices dampen the demand for overall energy in the newly industrialized fossil fuel-intensive countries. Consequently, as in the case of Bangladesh, it can be hypothesized that positive oil price shocks could possibly make way for greater renewable energy utilization in those newly industrialized countries as well. Finally, the short-run elasticity estimates reveal no correlation between CO_2_ emissions and REO shares in Bangladesh. Furthermore, the negative sign and statistical significance of the estimated lagged ECT shows that any deviation from the long-run equilibrium is corrected at a rate of 78.1% in the next period.

As far as the long-run elasticity estimates are concerned, it can be seen that FDI inflows despite dampening the REO shares in the short-run, tend to marginally elevate the shares in the long-run. A percentage rise in the inflows of FDI is predicted to enhance the REO shares by 0.05%, on average, *ceteris paribus.* Therefore, it can be said that in the long-run foreign finance in the form of FDI mitigates the constraints to producing power from renewables on a mass scale and also inhibits non-renewable electricity generations in Bangladesh. This could be envisioned as the technological spillover effects of FDI on the energy sector of Bangladesh which could be effective in developing renewable electricity in the national energy mix. This finding matches the conclusions made by Azam et al*.* (2015) for Southeast Asian countries. These studies have asserted that incoming FDI promotes the use of clean energy resources which can be linked to higher REO in the long-run. In contrast, Lin et al. (2016) highlighted the adverse long-run impacts of inward FDI on the REO shares of China. Thus, in line with both the short and long-run estimates of the FDI inflow elasticities of REO shares, it is recommended that the government attracts clean FDI into the relatively greener industries in Bangladesh. More importantly, channeling FDI towards the energy sector with the uplifting the quality of the nation’s energy infrastructure could be expected to amplify the REO shares further.

On the other hand, technological advancement, as indicated by lower energy intensity levels, is also found to enhance the REO shares in the long-run. Hence, it can be asserted that the positive impacts of technological progress on the prospects of enhancing the REO shares in the short-run are sustained over the long-run as well. A percentage fall in the energy intensity level is predicted to account for 0.82% higher shares of renewable electricity in aggregate electricity outputs of Bangladesh. These results match the similar opinions put forward by Murshed (2021d) in the context of Bangladesh and five other South Asian nations. Moreover, the relatively higher magnitude of the long-run elasticity estimate, in comparison to that in the short-run, implicates that persistent advancement of the technological stock can be asserted to progressively enhance the REO shares as well. Thus, it is ideal to invest heavily in research and development purposes as a means of financing technological innovation in Bangladesh. Simultaneously, attracting FDI towards the energy sector can also be a potential mechanism to catalyze the rate of technological advancement in the economy; thus, intensifying the REO shares further.

Other results reveal that the REO-inhibiting impacts of economic growth in the short-run are reduced in the long-run which further certifies that economic empowerment, through increments in the national income levels, helps to gradually overcome the barriers that uphold mass-scale production of renewable electricity in Bangladesh. However, economic growth still does not ensure higher shares of REO in the country which is evident from the negative signing of the corresponding long-run elasticity estimate. A percentage rise in the real per capita GDP figures causes the long-run REO shares by 0.15%, on average, *ceteris paribus.* This finding opposes the findings by Murshed and Tanha (2020) in which the authors opined in favor of economic growth stimulating REO shares in the context of a panel of four South Asian net oil-importing nations. On the other hand, domestic capital investments unlike the case in the short-run are found to be incapable of explaining the variations in Bangladesh's long-run shares of REO. The statistical insignificance of the predicted elasticity parameter attached to GCF affirms this claim. Besides, the long-run elasticity estimates also show that international trade, in comparison to the short-run scenario, dampens the REO shares more in the long-run. A rise in the trade openness index by 1% is associated with a fall in the REO shares by 1.58%, on average, *ceteris paribus.* Hence, it is recommended that the Bangladesh government revisits its foreign trade policies and adopt appropriate policy measures to downsize the trade of goods and services that embody the use of electricity generated from conventional non-renewable energy resources. Simultaneously, the government is expected to incentivize the exporting industries, in particular, to independently generate a certain amount of electricity using renewable resources rather than solely being dependent on in-grid fossil fuel-fired electricity supplies. This finding is comparable to the remarks by Murshed (2020b) in the context of the lower-middle-income countries including Bangladesh; the author claimed that higher openness to international trade reduced the renewable energy consumption shares.

The long-run impacts of oil price shocks are similar to the corresponding short-run impacts. A 1% rise in the real crude oil prices are seen to enhance the REO shares by 4.3% on average which, in comparison to the comparatively lower impacts in the short-run scenario, implies that persistent positive shocks to oil prices in the international markets facilitate the replacement of fossil fuels by the renewable alternatives concerning electricity generation purposes in Bangladesh. This particular result condemns the findings put forward by Murshed and Tanha (2020) in which the authors, using panel data estimation methods, found that increments in crude oil prices monotonically dampened the REO shares in Bangladesh, India, Pakistan and Sri Lanka. In line with both the short and long-run estimates of the oil price elasticity of REO found in this study, it can be claimed that substantial hikes in the world crude oil prices would ultimately eliminate Bangladesh’s imported oil-dependency for electricity generation purposes; thus possibly elevating the nations REO shares in the future. This is a key finding in the sense that oil prices in the international markets have currently rock bottomed, and turned negative, courtesy of the global coronavirus (COVID-19) pandemic which, in turn, could trigger greater imports of crude oil at extremely low prices. Consequently, the nation's dismal REO shares could well be at stake of declining further in the post-pandemic period. Hence, the government has to keep this concerning issue into consideration and adopt appropriate to refrain from importing crude oils in bulk. Finally, the long-run elasticity estimates also reveal that CO2 emission, although not being able to enforce the transition from non-renewable to renewable electricity generation in Bangladesh, is capable of elevating the REO shares in the long-run. The corresponding statistically significant elasticity estimate suggests that a 1% rise in the per capita CO2 emissions, in the long-run, is associated with a 0.49% rise in the REO shares, on average, *ceteris paribus.* Hence, it can be said that apprehensions concerning the CO2 emissions-induced climate change adversities could spark urgency for the government to incentivize mass-scale production of electricity using the environmentally-friendly primary renewable energy inputs; hence, gradually uplift the nation’s REO shares in the aggregate electricity outputs.

The short and long-run elasticity estimates, from the ARDL approach, in the context of model (2) are reported in Table 4. The short-run elasticities indicate that inflow of FDI adversely impacts the environmental quality in Bangladesh; hence the results verify the authenticity of the pollution haven hypothesis. The statistical significance and positive signs of the predicted elasticity parameters attached to the current and lagged levels of FDI certify this claim. However, the negative environmental impacts are seen to cease with time since the magnitude of the elasticity estimates at the current level of FDI inflows is relatively smaller than that at the lagged levels of FDI inflows. Therefore, once again it can be said that, with time, the relatively cleaner FDI flow into the Bangladesh economy; thus, the marginal increments in the ecological footprints tend to decline simultaneously. A similar positive correlation between FDI inflow and ecological footprints was highlighted in the context of the United States by Zafar et al. (2019). Besides, the short-run elasticity estimates confirm the authenticity of the EKC hypothesis to validate the inverted-U-shaped association between economic growth and ecological footprints in Bangladesh. The positive and negative signs of the elasticity parameters attached to the current and lagged levels of per capita GDP and its squared term, respectively affirm this claim. These findings imply that at the initial stages of economic growth, there is a trade-off between economic and environmental welfare which seems to diminish at higher per capita GDP levels. The short-run validation of the EKC hypothesis was also reported in the study by Hassan et al. (2019) for Pakistan. Among the other short-run determinants of ecological footprints in Bangladesh, urbanization is found to dampen environmental quality in the short-run. A percentage increase in the urbanization rate at its current and one-period lagged levels boosts the ecological footprints figures by 0.34% and 0.60%, on average, *ceteris paribus.* Thus, these results provide statistical support to the unplanned urbanization-induced environmental woes of Bangladesh. Nathaniel et al. (2019) also found a similar short-run association between urbanization and ecological footprints in the context of South Africa. Besides, the negative sign and statistical significance of the estimated lagged ECT in the context of model (2) shows that any deviation from the long-run equilibrium is corrected at a rate of 65.9% in the next period.

**Table 4:** The short and long-run elasticity estimates from the ARDL approach for model (2)

Dep. variable: lnEFP			
Short-run analysis		Long-run analysis	
∆(EFP (− 1))	− 1.042 (0.672)	lnFDI	0.273*** (0.112)
∆(lnFDI)	0.353*** (0.043)	lnGDPPC	41.641*** (10.221)
∆(lnFDI (− 1))	0.435** (0.217)	lnGDPPC^2^	− 3.051*** (1.012)
∆(lnFDI (− 2))	0.499** (0.249)	lnREO	− 1.654*** (0.390)
∆(lnGDPPC)	101.223*** (32.390)	lnREO*lnFDI	− 0.669** (0.335)
∆(lnGDPPC (− 1))	135.213*** (43.122)	lnEI	0.163** (0.082)
∆(lnGDPPC^2^)	− 34.081*** (8.224)	lnOPEN	0.157 (0.110)
∆(lnGDPPC^2^ (− 1))	− 12.112** (6.500)	lnOIL	− 0.188** (0.093)
∆(lnREO)	2.159 (2.080)	lnURB	0.121*** (0.022)
∆(lnREO (− 1))	0.843 (0.622)	BY1	− 3.623** (1.311)
∆(lnREO*lnFDI)	− 0.215 (0.181)	BY2	− 1.950*** (0.414)
∆(lnREO*lnFDI (− 1))	− 0.098 (0.072)	BY3	2.234** (1.112)
∆(lnEI)	− 1.367 (0.890)	BY4	− 3.121*** (1.341)
∆(lnOPEN)	3.501 (2.812)	BY5	− 1.623*** (0.412)
∆(lnOPEN (− 1))	− 1.861 (1.221)	Adj. R^2^	0.869
∆(lnOIL)	− 1.223 (1.010)	Obvs	45
∆(lnURB))	0.341*** (0.100)	Diagnostics	
∆(lnURB (-1))	0.602** (0.300)	BGodfrey	0.410
∆(BY1)	− 3.712 (0.541)	ARCH	1.012
∆(BY2)	− 1.202** (0.551)	CUSUM	Stable
∆(BY3)	2.348*** (0.121)	CUSUMSQ	Stable
∆(BY4)	− 3.200*** (0.598)		
∆(BY5)	− 1.912*** (0.231)		
ECT_t-1_	− 0.659*** (0.212)		

The standard errors are reported within the parentheses; Optimal lag selection is based on the AIC;*** and ** denote statistical significance at 1% and 5% significance levels

On the other hand, the long-run elasticity estimates reported in Table 4 shows that the negative impacts of FDI inflows on the environment in Bangladesh are sustained in the long-run. The statistical significance and positive sign of the long-run elasticity parameter attached to FDI inflows affirm this claim. A rise in the volume of FDI inflows by 1% in the long-run is seen to increase the ecological footprints figures on average by 0.27%, *ceteris paribus.* Therefore, it can be said that the ‘*pollution haven hypothesis*’ is a long-term problem for the Bangladesh economy. However, it is to be noted that the magnitude of the long-run elasticity is relatively smaller than the corresponding short-run elasticities which, to some extent, implies that FDI inflows tend to have low technological spillover effects whereby the positive impacts on the environment are not so pronounced; rather the damages are slightly less. This is a concerning finding in the context of Bangladesh which warrants restructuring of the nation’s foreign financing and financial globalization policies. The nation is better-off attracting cleaner FDI and restricting the inflows of the relatively dirtier ones. This finding of the adverse environmental impact of FDI inflow in Bangladesh corroborates the conclusions made in the study by Doytch (2020) in which the author claimed the ‘*pollution haven hypothesis*’ usually hold for the developing countries since the dirty FDIs tend to flow into these countries to exploit their weak environmental laws; this scenario is pretty similar to the case of Bangladesh where little emphasis is put on enforcing strict environmental acts to safeguard the environmental attributes. The long-run validation of the pollution haven hypothesis was also put forward by Khan et al. (2019) for 54 BRI countries including Bangladesh. Besides, the long-run elasticity estimates also confirm the validity of the EKC hypothesis for Bangladesh. The corresponding elasticity estimates reveal that initially a 1% rise in the long-run per capita GDP figures is associated with a rise in the ecological footprints by 41.64%, but in the latter stages of growth, the marginal effect seems to reduce the ecological footprints by 3.05% on average, *ceteris paribus.* Hence, it can be said that economic growth is both the cause and the long-run solution to the environmental problems of Bangladesh. The results are parallel to the conclusions made by Altıntaş and Kassouri (2020) and Destek and Sarkodie (2019) for 14 European and 11 industrialized economies, respectively; while these results contradict the assertions made by Ozcan et al. (2018) in the case of Turkey.

More importantly, the long-run analysis shows that changes in the REO shares determine environmental well-being in Bangladesh. A percentage rise in the REO shares in the aggregate electricity outputs of Bangladesh is found to reduce the ecological footprints by 1.65%, on average, *ceteris paribus.* Hence it can be said that RET, as indicated by a rise in the share of renewables in total energy consumption volumes, is a long-run phenomenon that, although is ineffective in improving environmental quality in the short-run, safeguards the long-run environmental sustainability goals of Bangladesh. This finding is comparable to the claims made by Ulucak and Khan (2020) where the authors opined that renewable energy use, natural resource rent, and urbanization curb ecological footprints in Brazil, Russia, India, China, and South Africa.

Another key finding from the long-run analysis shows that FDI inflows, despite directly damaging the environmental well-being in Bangladesh, have an indirect favorable outcome on the environment. The statistical significance and positive sign of the elasticity parameter attached to the interaction term between FDI inflows and REO shares suggest that both these macroeconomic aggregates jointly work to reduce the ecological footprints and, therefore, restore environmental harmony in Bangladesh. Thus, it is ideal for the nation to attract cleaner FDI and, more appropriately, channel the foreign funds towards the energy sector, particularly for the development of its renewable energy sector. Greater foreign investments in the energy sector are likely to induce technological spillover to overcome the major limitations impeding RET in Bangladesh. The pertinence of technological advancement concerning RET and environmental betterment can also be rationalized from the positive sign of the statistically significant elasticity parameter attached to the energy use intensity variable. The elasticity estimate shows that in the long-run a 1% fall in the energy intensity levels, which can be interpreted as a rise in the energy efficiency levels due to technological advancement, in particular, reduces the ecological footprints by 0.16%, on average, *ceteris paribus.* Therefore, inflows of the cleaner FDI can also be expected to have an indirect impact on environmental betterment provided it facilitates technological spillovers within the host economy of Bangladesh.

Furthermore, exogenous positive shocks to world crude oil prices are found to reduce the ecological footprints of Bangladesh in the long-run. A percentage increase in the real prices of crude oil is found to curb the ecological footprints by 0.19%, on average, *ceteris paribus.* A plausible explanation to this finding could be put forward in the sense that rising crude oil prices are likely to induce the RET phenomena, also supported by the corresponding elasticity estimates found in the context of model (1), whereby replacing the conventionally consumed fuels by the renewable alternatives can mitigate environmental deterioration in Bangladesh. Similar conclusions were made by Murshed and Tanha (2020) in the context of four South Asian economies including Bangladesh. Finally, the long-run estimates also certify that the short-run adverse impacts of unplanned urbanization on the environmental quality of Bangladesh are sustained over the long-run as well. This is parallel to the finding by Shahzad et al. (2020) for the developing countries and by Nathaniel et al. (2019) for South Africa.

Therefore, the overall findings from the regression analysis are in line with the three hypotheses put forward in this study. As a result, it can be claimed that FDI inflows play a key role in initiating the RET phenomena in Bangladesh but do not guarantee environmental improvement as a whole. A possible explanation behind these inconsistent findings could be the fact that environmental degradation in Bangladesh is not merely confined in terms of air pollution. Rather multiple aspects collectively contribute to deteriorate the nation's environmental quality. Hence, these findings once again highlight the multidimensionality of environmental problems faced by Bangladesh. Since RET is directly concerned with the mitigation of greenhouse gas emissions, it may not be sufficient to reduce the other forms of environmental hardships. Hence, policies should be undertaken to address this issue and try to utilize the foreign funds to also tackle the other dimensions of environmental adversity in Bangladesh. Moreover, since the hypothesis regarding a joint environmental impact of FDI inflows and REO was verified, Bangladesh must attract renewable energy technology development-related FDI. Consequently, both the energy security and environmental sustainability issues can be accounted for in tandem.

The findings from the diagnostic tests, as shown in Tables 2 and 3 for model (1) and model (2) respectively, suggest that both the regression models considered in this study are not subject to autocorrelation and heteroscedasticity issues. Moreover, the stability of ARDL-ECM elasticity estimates is confirmed by the CUSUM and CUSUMSQ charts.^2^ For robustness check, the long-run elasticities are re-estimated using the Fully Modified Ordinary Least Squares (Phillips & Hansen, 1990) and the Dynamic Ordinary Least Squares (Stock & Watson, 1993) regression techniques. The corresponding results, reported in Table 9 in the appendix, conform to the ARDL long-run elasticity estimates in terms of predicted signs; thus the robustness of the long-run elasticity estimates across different regression methods is affirmed. The causality investigations follow the regression analysis.

### Causality Results

Table 5 reports the results from the causality analysis for both model (1) and (2). In general, the results denote robustness across different causality estimation techniques which can be perceived to the identical causality estimates found from both the HH (2012) and TY (1995) tests. In the context of model (1), a unidirectional causality is found to be running from FDI inflows to REO shares in the long-run. Hence, in line with the corresponding elasticity estimates in the context of model (1), it can be said that attracting clean FDI into the Bangladesh economy can be effective in facilitating the RET process; thus, the nation's predominant reliance on fossil fuels for power generation purposes can gradually be phased out to integrate renewable electricity into the energy sector of Bangladesh. Therefore, channeling foreign investment funds towards the energy sector, particularly for the development of renewable power plants, should be a prioritized policy agenda of the government. The results are parallel to the findings in the study by Ahmad et al. (2019) for China. Besides, energy intensity levels are also found to causally influence the REO shares without feedback. Hence, it is once again assured that technological innovation, through a reduction in the intensity of energy use, is a pre-requisite to undergoing RET in Bangladesh. Song et al. (2019) also emphasized on the importance of technological progress for green innovation and sustainable resource management within the economy. This finding also implicates that FDI inflow-led technological innovation in Bangladesh can further enhance the nation’s REO shares. Among the other causal impacts concerning the REO shares in Bangladesh, the statistical significance of the test statistics certify between REO shares and per capita GDO, between REO shares and real crude oil prices, and between REO shares and CO_2_ emissions. These, in line with the corresponding elasticity findings, collectively imply that not only do rising national income level, crude oil prices and CO_2_ emissions facilitate the RET phenomenon, the higher shares of renewable electricity in the aggregate electricity outputs also determine economic growth, neutralize the adversities of oil-price volatilities and environmental welfare in Bangladesh.

**Table 5:** Hacker and Hatemi-J Bootstrap (2012) and Toda and Yamamoto (1995) causality test results

Model (1)			Model (2)		
Null hypo.	HH (2012)	TY (1995)	Null hypo.	HH (2012)	TY (1995)
	MW Test Stat	W Test Stat		MW Test Stat	W Test Stat
lnFDI ≠ lnREO	11.228***	5.223***	lnFDI ≠ lnEFP	6.170**	3.604***
lnREO ≠ lnFDI	1.828	0.212	lnEFP ≠ lnFDI	1.788	0.604
lnEI ≠ lnREO	9.223***	2.938***	lnGDPPC ≠ lnEFP	8.879***	3.184***
lnREO ≠ lnEI	1.899	1.212	lnEFP ≠ lnGDPPC	9.771***	5.220***
lnGDPPC ≠ lnREO	10.221***	6.212***	lnREO ≠ lnEFP	8.828***	3.389**
lnREO ≠ lnGDPPC	9.289***	5.829***	lnEFP ≠ lnREO	1.108	0.670
lnGCF ≠ lnREO	1.214	0.782	lnEI ≠ lnEFP	5.297**	2.229*
lnREO ≠ lnGCF	1.209	0.711	lnEFP ≠ lnEI	1.605	1.019
lnOPEN ≠ lnREO	1.665	1.112	lnOPEN ≠ lnEFP	1.402	1.120
lnREO ≠ lnOPEN	1.808	1.132	lnEFP ≠ lnOPEN	2.012	1.814
lnOIL ≠ lnREO	12.228**	6.212***	lnOIL ≠ lnEFP	7.909***	3.289**
lnREO ≠ lnOIL	7.269***	3.210**	lnEFP ≠ lnOIL	1.299	1.116
LnCO_2_ ≠ lnREO	10.218***	3.329**	lnURB ≠ lnEFP	9.368***	3.118**
lnREO ≠ lnCO_2_	12.219***	4.219***	lnEFP ≠ lnURB	1.209	1.024

** ≠ **denotes does not Granger causes; HH refers to the Hacker and Hatemi-J bootstrap (2012) and the TY refers to the Toda and Yamamoto (1995) causality tests; MW and W refer to the modified Wald and the Wald test statistics; The modified Wald statistics are estimated using bootstrap approach; ***, ** and * denote statistical significance at 1%, 5% and 10% significance levels, respectively

On the other hand, the causality estimates in the context of model (2) also depict unidirectional causation, without the feedback, from FDI inflows to ecological footprints in Bangladesh. This finding, along with the positive sign of the corresponding elasticity estimate concerning FDI inflows and ecological footprints, implicates that the quality of the FDI flowing into the Bangladesh economy does not safeguard the environmental goals of the nation. Thus, it is pertinent to restructure the foreign finance policy of the government whereby emphasis must be given to attract the relatively cleaner and renewable energy-intensive forms of FDI. The unidirectional causal finding contradicts the bidirectional association between FDI inflows and ecological footprints reported by Khan et al. (2020) for China, India, and Pakistan. Moreover, unidirectional causality from REO shares to ecological footprints is also ascertained from the causality analysis. This, in line with the corresponding elasticity estimate, implies that enhancing the REO shares to phase out the fossil fuel dependency is a plausible solution to the environmental hardships in Bangladesh. Similarly, Sharif et al. (2020) found unidirectional causality stemming from renewable energy use to ecological footprints in the case of Turkey. Besides, unidirectional causation from energy use intensity levels to ecological footprints is also revealed. Therefore, this finding further asserts the need for technological innovation, synonymous with a decline in the energy use intensity, for mitigating the ecological footprints figures in Bangladesh. Moreover, shocks to real crude oil prices are also estimated to causally influence the ecological footprints. In line with the corresponding elasticity estimate, this unidirectional causation between oil price and ecological footprints suggests that higher oil prices induce the substitution of fossil fuels by renewable alternatives for electricity generation processes which, in turn, can effectively reduce the ecological footprints levels in Bangladesh. Furthermore, the causality estimates also predict a unidirectional causality running from urbanization to ecological footprints which, along with the corresponding elasticity estimate, highlights the adverse environmental impacts associated with the unplanned urbanization problems in Bangladesh. Similar causality in the context of G7 countries was also stated in the study by Ahmed et al. (2020). On the other hand, a feedback effect between per capita GDP and ecological footprints is also witnessed. This implies that affluence plays a key role in influencing the environmental indicators in Bangladesh. Moreover, higher levels of national income, in turn, can ideally empower the Bangladesh economy to control environmental pollution to a large extent. Identical bidirectional causation between GDP per capita and ecological footprints in the context of Turkey was opined by Ozcan et al. (2018).

## Conclusion

Following the global appeal to undergo RET for ensuring energy security and environmental sustainability, the world economies have reached a consensus to align their respective economic growth strategies with the environmental development welfare-maximizing targets. However, undergoing RET is conditional on overcoming several constraints that have traditionally impeded the RET phenomena, especially across the underdeveloped economies. Among these, technological backwardness and infrastructural underdevelopment are hypothesized to be the major limitations hampering the integration of renewables into the energy sectors of these nations. Against this backdrop, this study evaluated how potential technological spillovers from FDI inflows can overcome these constraints to facilitate RET in Bangladesh. The central focus of this study was to unearth the dynamic associations between FDI inflows and REO shares in Bangladesh and to also investigate the impacts, both direct and indirect, of such FDI on the nation’s environmental quality quantified in terms of the ecological footprints.

The results from the regression and causality analyses, in a nutshell, implied that foreign direct investments in the long-run enhance the REO shares in Bangladesh. On the other hand, FDI inflows were also found to directly attribute to environmental degradation through boosting the nation’s levels of ecological footprints since the ‘*pollution haven hypothesis*’ was validated by the corresponding elasticity estimates. However, interestingly, a joint favorable environmental impact of higher FDI inflows and greater REO shares was also evidenced. Hence, these contrasting findings imply that the nature of the FDI determines its impact on RET and environmental sustainability. Accordingly, several policy recommendations are suggested. Firstly, it is pertinent for the government to channel foreign investments for the development of the renewable energy sector which can simultaneously foster the nation's energy security and environmental sustainability objectives. Besides, it is also necessary for Bangladesh to attract FDIs that have the potential of exerting knowledge spillover impacts for enabling large-scale renewable electricity generation in the country. In this regard, the utilization of foreign finance for the development of the domestic energy infrastructure can also be anticipated to further expedite RET in Bangladesh. At the same time, since FDI inflows were found to degrade the environment by boosting the ecological footprints, it is equally important for the government to restructure the national financial globalization policies to monitor and restrict the inflows of dirty FDI. Thus, it is compulsory for the nation to enhance the stringency of the existing environmental rules and regulations in order for Bangladesh to safeguard its economy from transforming into a pollution haven.

Secondly, since the long-run elasticity estimates and the causality findings statistically authenticated the EKC hypothesis in Bangladesh, it is recommended that the nation continues to focus on expanding the size of its economy without being fearful of compromising the environmental attributes in the process. This is because the validation of the EKC hypothesis implies that economic growth is both the cause and the panacea to the environmental hardships faced across Bangladesh. However, the growth strategies are ideally to be themed on the use of renewable energy resources in order to gradually phase out the nation's fossil fuel dependency and simultaneously restore the environmental balance. Additionally, it is extremely important for Bangladesh to establish sustainable consumption and production process which would further facilitate the prospects of having complementarity between economic and environmental well-being. Lastly, since the results portrayed that unplanned urbanization is detrimental to Bangladesh’s environmental sustainability, it is suggested that the government adopts appropriate policies to ease out the nations’ unplanned urbanization woes. In this regard, the relocation of the industrial activities from the urban to the rural areas can be helpful.

As part of the future scope of research, this study can be extended to specifically analyze the impacts of China's outward-FDI on the changes in the REO shares and ecological footprint figures of Bangladesh for unearthing key policy implications regarding the efficacies of allowing such foreign investments to take place. Furthermore, the recently introduced quantile ARDL technique for regression and causality analyses can be performed to assess the possible heterogeneity of the findings across different quantiles of the REO shares and ecological footprints levels of Bangladesh.

## Appendix

See Tables

**Table 6:** The definitions and data sources

Symbol	Definition	Source
lnREO	Electricity output generated from renewable resources (ratio of total electricity output)	Authors’ own calculation from World Development Indicators (World Bank, 2020)
lnEFP	Total consumption ecological footprints per capita (global hectares of land)	Global Footprint Network (GFN, 2019)
lnFDI	Net foreign direct investment inflows (US dollars)	World Development Indicators (World Bank, 2020)
lnRGDPPC	Gross Domestic Product (constant 2010 US dollars)	World Development Indicators (World Bank, 2020)
lnGCF	Gross capital formation (constant 2020 US dollars)	World Development Indicators (World Bank, 2020)
lnCO2	Carbon intensity (kg per constant 2010 US dollars)	World Development Indicators (World Bank, 2020)
lnOPEN	Trade openness (sum exports and imports as a ratio of GDP)	World Development Indicators (World Bank, 2020)
lnOIL	Spot crude oil prices (constant 2016 US dollars per barrel)	Statistical Review of World Energy 2019 (British Petroleum)
lnURB	Urbanization rate (Urban population as a ratio of total population)	World Development Indicators (World Bank, 2020)
lnEI	Energy intensity (kg of energy consumed per constant 2010 US dollars)	World Development Indicators (World Bank, 2020)

6,

**Table 7:** The descriptive statistics

Variables	Mean	Std. Dev	Min	Max	Skew	Kurt
lnREO	4.33	2.88	1.12	11.43	0.92	2.82
lnEFP	18.8	0.3	18.4	19.29	0.13	1.7
lnFDI	19.06	2.45	14.15	21.94	− 0.68	2.2
lnRGDPPC	6.4	0.29	5.99	6.88	0.27	1.69
lnGCF	23.6	0.65	22.57	24.54	− 0.08	1.73
lnCO2	− 0.82	0.11	− 1.02	− 0.64	− 0.63	2.24
lnOPEN	3.47	0.29	2.94	3.87	− 0.25	1.91
lnOIL	3.88	0.55	2.93	4.78	0.29	1.84
lnURB	3.27	0.19	2.99	3.58	0.17	1.72
lnEI	4.45	0.06	4.32	4.54	− 0.29	2.05

^7^,

**Table 8:** The correlation matrix

	lnREO	lnFDI	lnRGDPPC	lnGCF	lnCO2	lnOPEN	lnOIL	lnURB	lnEFP	lnEI
lnREO	1									
lnFDI	0.70	1								
lnRGDPPC	0.74	0.79	1							
lnGCF	0.97	0.58	0.64	1						
lnCO2	0.69	0.74	0.75	0.63	1					
lnOPEN	0.86	0.53	0.53	0.46	0.55	1				
lnOIL	0.71	0.80	0.79	0.60	0.74	0.52	1			
lnURB	0.62	0.64	0.65	0.54	0.63	0.55	0.62	1		
lnEFP	0.79	0.82	0.84	0.85	0.74	0.83	0.75	0.82	1	
lnEI	0.88	0.63	0.64	0.54	0.78	0.81	0.70	0.72	0.82	1

^8^,

**Table 9:** The FMOLS and DOLS regression results in the context of models (1) and (2)

Model (1)			Model (2)		
Dep. Var.: lnREO			Dep. Var.: lnEFP		
Estimator	FMOLS	DOLS	Estimator	FMOLS	DOLS
Regressors			Regressors		
lnFDI	− 1.733** (0.867)	− 0.745*** (0.001)	lnFDI	0.442*** (0.121)	0.262*** (0.101)
lnGDPPC	3.031*** (0.054)	3.221*** (0.043)	lnGDPPC	49.051*** (12.224)	51.019*** (13.222)
lnGCF	− 4.610*** (0.042)	− 3.896** (1.949)	lnGDPPC^2^	− 3.236*** (1.012)	− 4.023*** (1.211)
lnOPEN	− 1.160** (0.580)	− 0.965*** (0.010)	lnREO	− 1.601** (0.800)	− 1.911*** (0.951)
lnOIL	0.457*** (0.004)	0.083** (0.041)	lnREO*lnFDI	− 0.299** (0.149)	− 0.485*** (0.190)
lnCO_2_	− 8.120*** (0.030)	− 4.011***	lnEI	0.170** (0.851)	0.182*** (0.312)
BY1	0.329*** (0.101)	0.412** (0.208)	lnOPEN	0.159 (0.121)	0.150 (0.100)
BY2	0.400** (0.199)	0.431*** (0.161)	lnOIL	− 0.169*** (0.031)	− 0.196*** (0.040)
BY3	− 0.140*** (0.041)	− 0.152*** (0.034)	lnURB	0.157*** (0.029)	0.144** (0.719)
BY4	− 1.421** (0.710)	− 1.512*** (0.451)	BY1	− 3.024***	− 3.424***
BY5	0.301*** (0.100)	0.350*** (0.101)	BY2	− 1.476***	− 1.376***
			BY3	2.142***	2.344***
			BY4	− 3.225***	− 3.105***
			BY5	1.707***	1.690***

FMOLS = Fully Modified Ordinary Least Squares regression; DOLS = Ordinary Least Squares regression; The standard errors are reported within the parentheses; The optimal lag selection is based on AIC; *** and ** denote statistical significance at 1% and 5% levels, respectively

9.

## Footnotes

1 For brevity, the VIF output is not reported but can be made available upon request.

2 For brevity, the charts are not reported but can be supplied upon request.
